# Current Preventions and Treatments of aGVHD: From Pharmacological Prophylaxis to Innovative Therapies

**DOI:** 10.3389/fimmu.2020.607030

**Published:** 2020-12-17

**Authors:** Sina Naserian, Mathieu Leclerc, Sara Shamdani, Georges Uzan

**Affiliations:** ^1^INSERM UMR-S-MD 1197, Hôpital Paul Brousse, Villejuif, France; ^2^Paris-Saclay University, Villejuif, France; ^3^CellMedEx, Saint Maur Des Fossés, France; ^4^Service d’Hématologie Clinique et de Thérapie Cellulaire, Hôpital Henri Mondor, Créteil, France; ^5^INSERM U955, Institut Mondor de Recherche Biomédicale, Créteil, France; ^6^Faculté de Médecine de Créteil, Université Paris-Est, Créteil, France

**Keywords:** hematopoietic stem cell transplantation, graft versus host disease, T cells, immunoregulation, tolerance induction, cell therapy, TNFα-TNFR2 signaling pathway

## Abstract

Graft versus host disease (GVHD) is one of the main causes of mortality and the reason for up to 50% of morbidity after hematopoietic stem cell transplantations (HSCT) which is the treatment of choice for many blood malignancies. Thanks to years of research and exploration, we have acquired a profound understanding of the pathophysiology and immunopathology of these disorders. This led to the proposition and development of many therapeutic approaches during the last decades, some of them with very promising results. In this review, we have focused on the recent GVHD treatments from classical chemical and pharmacological prophylaxis to more innovative treatments including gene therapy and cell therapy, most commonly based on the application of a variety of immunomodulatory cells. Furthermore, we have discussed the advantages and potentials of cell-free therapy as a newly emerging approach to treat GVHD. Among them, we have particularly focused on the implication of the TNFα-TNFR2 axis as a new immune checkpoint signaling pathway controlling different aspects of many immunoregulatory cells.

## Introduction

Bone marrow transplantation (BMT), also called hematopoietic stem cell transplantation (HSCT), is a process of infusing stem cells taken from healthy donors into recipient patients. Though initially developed to treat damage caused by exposure to high doses of radiation, today allogeneic HSCT is the treatment of choice for many blood malignancies such as acute leukemias, myelodysplastic syndrome and lymphomas (1), and inherited or acquired non-malignant blood disorders, such as sickle-cell anemia and aplastic anemia (2, 3).

In allogeneic HSCT, patients first receive a conditioning regimen consisting of combination chemotherapy sometimes associated with radiotherapy and T-cell-depleting antibodies. Patient conditioning is followed by the infusion of donor HSCs which could be harvested from the bone marrow (BM) or, more commonly nowadays, from the peripheral blood (PB) of donors that have been treated with granulocyte colony-stimulating factor (G-CSF) to induce the release of immature hematopoietic progenitors into the circulation. BM cells and G-CSF-mobilized peripheral blood stem cells (PBSCs) are both enriched in hematopoietic progenitors; however, they also contain mature CD4^+^ and CD8^+^ T cells. In general, donor T cells present in the graft are essential for three main purposes: 1) They are involved in hematopoietic engraftment (4). 2) Reconstitution of T cells immunity (particularly in adults with reduced thymic function, i.e. the majority of transplanted patients, as recipients’ age has significantly increased over the last 2 decades) (5). 3) Mediating a potent beneficial antitumor effect, known as graft versus leukemia/tumor effect (GVL/GVT) (6).

Unlike solid organ transplantation, the main reason to apply HSCT is not only to replace a non-functioning tissue, but to benefit from a strong GVL/GVT effect. About 60 years ago, Barnes and Loutit suggested that BM transplantation was associated with an anti-tumor effect that could not be explained by pre-transplantation chemotherapy or irradiation (7). Furthermore, Butturini showed the loss of anti-tumor effect after T cell depletion (8). The first precise work focused on GVL effect was conducted by Horowitz et al., on a sample of 2,254 patients who received BM graft. Horowitz demonstrated that the relapse risk of leukemia was correlated with the occurrence of GVHD, mostly in its chronic presentation; i.e. those patients developing chronic GVHD had a lower risk of relapse as compared with patients developing only acute GVHD or no GVHD at all. On the other hand, the highest risk of leukemia relapse was observed among recipients of T cell-depleted grafts or in case of a syngeneic donor (6). In parallel, the concept that allogeneic cells have an anti-leukemia effect independent of GVHD is supported by studies on mice, where T cells with GVL but not GVHD activity were identified (9). This supports the independency of GVHD from the GVL effect at least in mouse models. Today we clearly know that these effects result from the recognition of residual malignant host tumor cells and other non-malignant residual cells by alloreactive donor T cells within the graft. In addition, NK cells have been also shown to have anti-tumoral activities (10). Other studies gave rise to the hypothesis that NK cells attack targets that do not express ‘‘self’’ MHC class I molecules (11). Interestingly, due to the presence of killer cell immunoglobulin-like receptors (KIRs), MHC class I receptors, NK cells can distinguish between normal and tumoral cells and kill those that do not have MHC class I molecules specific for their KIRs (12).

Despite the beneficial effects, several serious complications might occur after HSCT. One of the principle causes of post-HSCT mortality is GVHD, which is also a major cause of morbidity in up to 50% of transplanted recipients (13).

Around 50 years ago, GVHD was initially reported by Barnes, Loutit, and Micklem as a ‘‘secondary disease of radiation chimera’’ and was classically defined by Billingham as a syndrome in which donor immunocompetent cells recognize and attack host tissues in immuno-compromised allogeneic recipients (14, 15). Billingham formulated three conditions for the development of GVHD:

1. The graft must contain immunologically competent cells. Mature T cells are the principle immunocompetent cells of the graft that are responsible for development of GVHD. Moreover, the severity of GVHD is directly correlated with the number of transfused T cells (16).
2. The recipient must express tissue antigens that are not present in the transplant donor. The incompatibility between donor’s and recipient’s tissues, in particular MHCs (Major Histocompatibility Complex), known in human as HLA (Human Leukocyte Antigen), is directly correlated with the incidence of GVHD (17). Today, thanks to a better understanding of the exact immunological bases of GVHD, we are sure that not only differences of MHCs, but also the diversity of minor histocompatibility antigens could cause this disease. In full HLA-matched allogeneic HSCT, minor H antigens disparities between donor and recipient are associated with severe GVHD (18, 19).
3. The patient must be incapable of rejecting the graft. Since the presence of alloreactive recipient T cells would cause the rejection of the allograft, recipients must primarily undergo immunosuppressive treatments.

In 2006, these old criteria have been revised with the addition of a fourth and essential condition: donor lymphocytes must be able to migrate and home to host target tissue of GVHD. T cell have the necessary combination of homing and chemokine receptors to interact with the endothelium at the target tissues (20).

As mentioned earlier, it is now clear that the main immunologically competent cells in the triggering of acute GVHD are donor T cells of the blood or bone marrow transplants (21). Generally, patients whose immune systems are suppressed and receive white blood cells from another individual are at high risk of developing the disease. However, GVHD can seldom develop in various clinical settings other than HSCT, such as solid organ transplantation when T cells within the donor’s tissues are not eliminated, or after transfusion of blood products (post-transfusion GVHD) (22–24).

In humans, GVHD is either acute (aGVHD), which classically occurs within 100 days of transplant, but can also develop later following reduced-intensity conditioning (RIC) regimens (“late-onset acute GVHD”) or chronic (cGVHD), which typically develops 100 days after transplantation (25–27). The mechanisms involved in these two manifestations are different; aGVHD demonstrates an exacerbated inflammatory mechanism, whereas cGVHD displays autoimmune features.

The development of GVHD and its severity in transplanted recipients depend on several factors like the donor/recipient HLA-matching, recipient’s age, genetic polymorphisms, toxicity of the conditioning regimen, stem cell source (bone marrow versus peripheral blood), donor/recipient sex pairs (higher risk for female donor into male recipient) and prophylaxis approach of GVHD (28). Classically, corticosteroids at the dose of 2 mg/kg/day are the first line treatment of established grade II or higher aGVHD, but patients with steroid-refractory aGVHD have a dismal outcome with long term mortality rates that historically reached 90% (29). In this review, we discuss in detail the current strategies of prophylaxis and treatments of GVHD. We have categorized these treatments into classical ones based mostly on pharmacological prophylaxis and innovative therapies such as gene, cell and immune therapy of aGVHD.

### Classical Pharmacological Prophylaxis

Despite our profound understanding of aGVHD at the molecular level, the limited successes of established immune therapies for prevention and treatment of GVHD remain unsatisfactory. This might be in turn due to the observed controversial effects of the majority of molecules involved in the pathophysiology of this disease, thus complicating the establishment of the best mechanism of prevention. The ideal clinical achievement in HSCT would be to extenuate harmful effects of donor T cells while preserving and accentuating GVL/GVT effect, a scenario that has not been completely yielded yet. Since the main cause of GVHD is the presence of donor T cells in the graft, most prophylaxes are focused on either inhibition or depletion of these T lymphocytes or induction of tolerance.

#### Inhibition of Alloreactive T Cells

**Figure 1:**
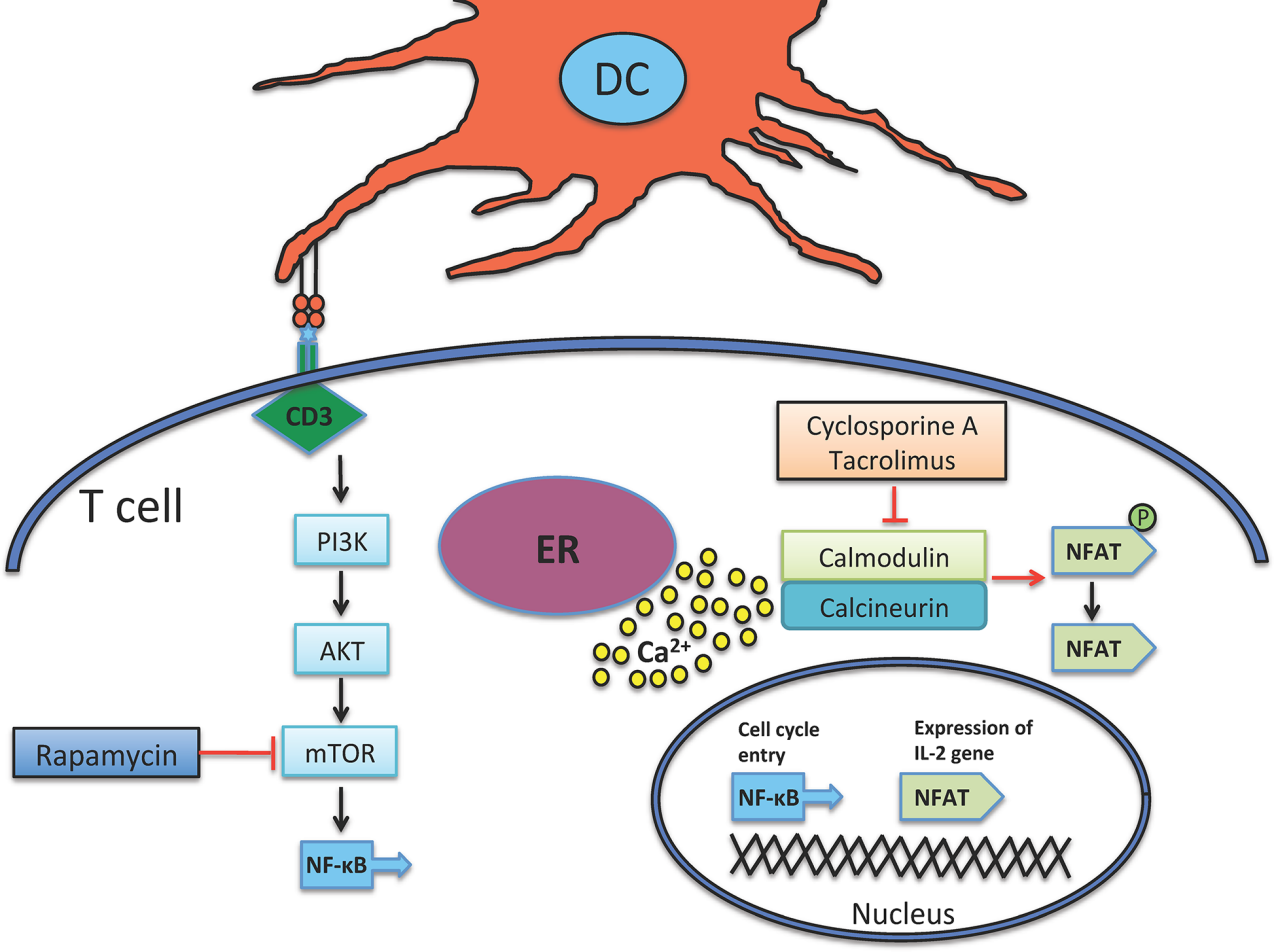
Mechanism of action of immunosuppressant agents. Both Cyclosporine and Tacrolimus inhibit calcineurin, a calcium-dependent phosphatase that dephosphorylates and further activates NFAT, which in turn provokes IL-2 production. Calcineurin is activated by liberated calcium from ER. mTOR is another target down-stream of CD3 signaling, it is activated by the PI3K enzyme. mTOR induces cellular division and is inhibited by Rapamycin. ER, endoplasmic reticulum.

In 1980s the introduction of two new immunosuppressive agents, Cyclosporine A and Tacrolimus, which prevent T cell activation *via* inhibiting calcineurin, significantly improved allograft survival rate. To work, they fix themselves on calcineurin-calmodulin-Ca2^+^ complex and inhibit the phosphatase activity of calcineurin, which in turn stops the translocation of nuclear factor of activated T cell (NFAT) and NF-κB into nucleus (**Figure 1**) (30–32), therefore, hamper the transcription/expression of IL-2 and IL-2 receptor (IL-2R or CD25).

The standard prophylaxis of GVHD is the combination of a calcineurin inhibitor with methotrexate, a drug that interferes with alloreactive T cells division (33). In the setting of unrelated donor transplantation, the addition of anti-thymocyte globulin (ATG) can reduce the incidence of both acute and chronic GVHD, without any significant increase in relapse risk (34). In the late 90’s, the advent of RIC regimens came with new “methotrexate-free” GVH prophylaxis protocols (35), such as the combination of cyclosporine and ATG (36), that can also be associated with mycophenolate mofetil, mainly in case of unrelated donor transplantation (37).

Sirolimus (rapamycin) is a molecule that forms a complex with mammalian target of rapamycin (mTOR), and therefore debars the PI3K-AKT-mTOR pathway and also that of NF-κB with the concomitant reduction of DNA transcription/translation, cell cycle progression and ultimately T cell suppression (**Figure 1**) (38). Rapamycin is highly used in solid organ transplantation (39, 40) and in autoimmune diseases like type 1 diabetes, which demonstrates that rapamycin not only depletes effector T cells but also enhances the expansion of regulatory T cells (Tregs) that can further suppress effector activity of T cells (41, 42). In case of GVHD, some clinical trials have shown its protective effect (43–45).

Despite partial achievements, none of the above-mentioned therapeutics could satisfactorily prevent GVHD, knowing that still 50% of transplanted patients show the disorder. Additionally, because all these agents are conferring a general immunodeficiency, they unfortunately interfere with the desired GVL effect (46).

In case of aGVHD occurrence, standard first-line treatment relies on high doses (2 mg/kg/day) of corticosteroids (47). Unfortunately, all attempts to improve on the curative treatment of established aGVHD have turned into repeating failures, either with strategies aiming at increasing the doses of steroids (48), or combining them with other drugs (49, 50). In case of steroid-refractory aGVHD, many second-line treatments have been tested, and until recently, none of them had demonstrated superiority over others, and thus no standard treatment was recognized in this setting (47). However, a recent phase III study has established ruxolitinib, an oral selective inhibitor of JAK1 and JAK2, as the most potent molecule in steroid-refractory aGVHD, with an acceptable safety profile, making it a new standard of care (51). The rationale for targeting JAK1/2 is the major role of its signaling in inflammation, tissue damage, T-cell activation, lineage commitment and survival, but also activation of neutrophils and differentiation and maturation of dendritic cells, all of which are involved in the pathogenesis of aGVHD (52–55).

#### Depletion of Alloreactive T Cells

The idea of depleting T cells from the infused cell product is not new and dates back to 1980s and 1990s; for such, three main strategies were considered effective: 1) *Ex-vivo* negative selection of T cells. 2) *Ex-vivo* positive selection of CD34^+^ stem cells. 3) In-vivo depletion of T cells by antibodies.

Heeding these strategies, total T cells removal from the graft resulted in reduced incidence and severity of aGVHD (56–58). Nevertheless, the presence of T cells in graft was demonstrated as very important, so their depletion caused poor hematopoietic engraftment, increased incidence of disease relapse and opportunistic infections (56, 59, 60). Later on, the invention of magnetic beads led to more accurate targeting and also more efficient depletion of T cells. Interestingly, three separate clinical trials, targeting CD3^+^T cells removal, CD3^+^T cells plus CD19^+^ B cells depletion, or αβ T cells plus CD19^+^ B cells elimination, ended in lower incidence of aGVHD and better engraftment rate (61–63).

Positive selection of CD34^+^ stem cells by magnetic beads is potentially an effective method to deplete alloreactive donor T cells prior to transplant which resulted in remarkable reduction of aGVHD and cGVHD (64–66). The major limitations of this method are increased risk of infections, which resulted in 40% mortality, and a high incidence of cancer recurrence.

**Figure 2:**
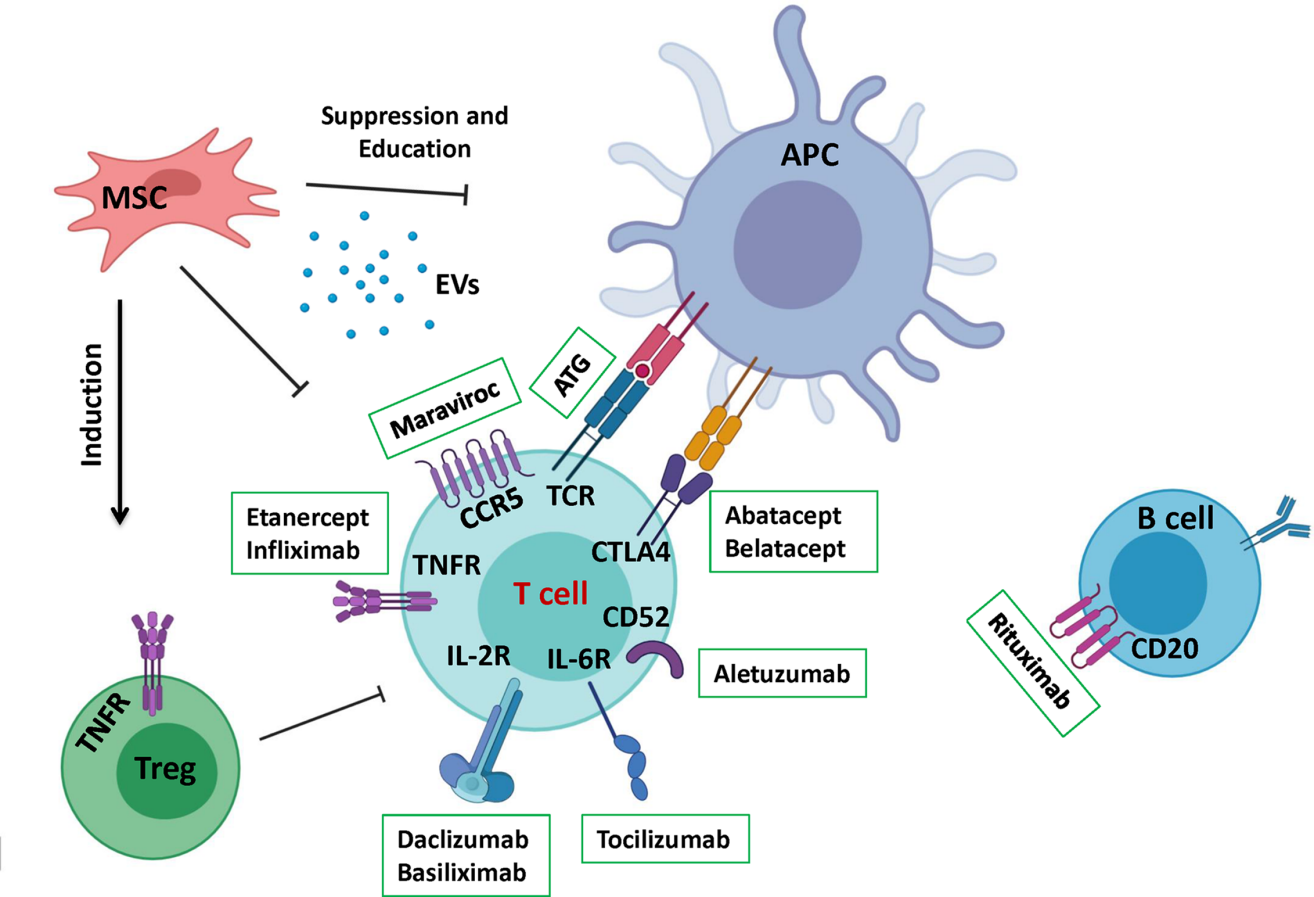
A summary of the different therapeutics applied in aGVHD treatment. This schematic depicts a summary of recent therapeutics that has been developed to control GVHD. Therapeutic approaches are divided to pharmacological drugs mostly aiming to target a signaling pathway or cellular therapy that renders vaster regulatory effects.

ATG is a polyclonal antibody preparation that triggers simultaneous in-vivo depletion of donor and host T cells *via* induction of apoptosis, which enables a better control of transplant rejection or GVHD occurrence (**Figure 2**) (67, 68). Although ATG seems more convenient for the purpose, its high doses were associated with increased infections (69). In addition, ATG affects B cells, NK cells and APCs, thus works as a non-specific targeting agent (70). In a recent consensus, ATG/ATLG (anti-T lymphocyte globulin) was strongly recommended as part of myeloablative conditioning regimen prior to matched or mismatched unrelated allogeneic HSCT to prevent both aGVHD and cGVHD. In reduced intensity or non-myeloablative conditioning regimens, ATG/ATLG was estimated appropriate to reduce the incidence of GVHD, but an increased risk of relapse was suggested to take into account (71).

Introduction of monoclonal antibodies made T cells depletion even more specific. T10B9 is a monoclonal antibody (mAb) which targets T cell receptor (TCR) αβ heterodimer region of CD3^+^T cells (72). A combination of this mAb and Cyclosporine A was compared with methotrexate and cyclosporine A treatment in a phase 2/3 clinical trial, and the results showed reduction in grades 3 to 4 aGVHD but a higher risk of chronic myelogenous leukemia relapse (73). Another example of this kind is Alemtuzumab (Campath), which targets CD52 antigen (**Figure 2**) expressed on the surface of T and B cells but not on CD34^+^ stem cells (74). Its first application was reported to reduce multiple sclerosis (MS) severity and relapse (75). A recent study was performed on 201 adult patients receiving a RIC allograft. With a median follow-up of 24 months, the cumulative incidences of aGVHD and late acute GVHD grades II-IV (grades III-IV) were 34% (13%) and 20% (8%) respectively. Furthermore, the cumulative incidences of cGVHD and overlap syndrome were 4% and 7% respectively (76). Although Alemtuzumab administration before HSCT, from related or unrelated donors, resulted in a lower incidence of GVHD, it could remain in the blood at lympholytic level for 1 to 2 months after transplantation. Consequently, the immune system reconstitution was considerably delayed, leading to a high incidence of viral infection and relapse (77).

More recently, the use of post-transplant cyclophosphamide (PT-Cy) has brought T-cell replete haplo-identical transplantation up to date, with remarkable results regarding GVHD incidence in this high-risk setting, thanks to the selective depletion of alloreactive T cells, while sparing regulatory T cells (78, 79). PT-Cy has also shown efficacy in transplantation with HLA-matched related and unrelated donors (80), and phase III clinical trials comparing PT-Cy and standard GVHD prophylaxis are currently ongoing (NCT03818334, NCT02345850).

Although the inhibition and depletion of alloreactive T cells are classically more studied to prevent GVHD, several other research works have been investigating on alternative strategies to block T cell migration towards GVHD target organs. This is in accordance with the more recently defined fourth criteria of GVHD development (20). A variety of molecules have been testing for this effect, notably maraviroc that blocks CCR5 (**Figure 2**) (81, 82), fingolimod (FTY720) that mostly interferes with T cells’ infiltration into skin (83–85), and natalizumab that has been shown to mediates homing of lymphocytes to the gastrointestinal tract (86), with promising results.

### Innovative Therapies

Current progress in biomedical research has opened the door for new innovative therapy approaches including gene and cell therapies. Gene transfer technologies, including the suicide gene approach, are promising tools to manipulate donor T cell immunity, to boost the GVL effect, to foster functional immune reconstitution, and to prevent or control GVHD. Cell therapy of aGVHD is based on distinctly ex-vivo or in-vivo expansion of Tregs, which are the natural immunosuppressant cells of the body. Moreover, the application of mesenchymal stromal cells (MSCs), regulatory macrophages, innate lymphoid cells (ILCs), NKT cells and endothelial progenitor cells (EPCs), based on their immunoregulatory and/or regenerative properties have also been, or are currently being investigated, showing very promising results.

### Gene Therapy of aGVHD

Gene therapy of aGVHD consists in transferring a suicide gene into donor T lymphocytes, which can be selectively controlled after transplant. Herpes simplex virus thymidine kinase (HSV-TK) has already been introduced as a cell-cycle dependent suicide gene (87, 88). In the presence of ganciclovir (GCV), an anti-herpes drug, infected cells catalyze the generation of triphosphate ganciclovir that further inhibits DNA chain elongation, which is toxic to proliferating cells (89, 90). In-vitro and in-vivo preclinical studies in mice (91, 92) and afterward phase I/II clinical trials have demonstrated that the retroviral-mediated transfer of HSV-TK suicide gene into donor T cells prior to graft infusion allows efficient control of donor T cell alloreactivity (93, 94). In the latter clinical trial, the investigators also showed that these infected T cells improve immune reconstitution and could provide GVL effect. However, there are some possible drawbacks for applying this strategy. In immuno-compromised patients, TK may lead to undesired elimination of transduced cell populations as a result of the immunogenicity of this viral protein. In addition GCV is a drug used to treat cytomegalovirus (CMV) infection which commonly affects immuno-compromised patients. Administration of GCV in CMV infected patients could result in undesired TK-cell killing (95). Also, as suggested in the study by Maury et al., elimination of TK^+^ cells after ganciclovir administration may not prevent GVHD caused by a putative in-vivo expansion of the small proportion of TK^-^ alloreactive T cells in this lymphopenic setting (94).

Another suicide gene that has also been tried in a phase I clinical trial, is inducible human caspase 9 (iC9), a hybrid protein consisting of a human FK506-binding protein (FKBP12) linked to a modified human caspase 9 lacking the caspase recruitment domain (CARD). This transgene can be activated by a single administration of a small-molecule drug (AP1903). Thanks to the accelerated immune reconstitution, patients have immediate and sustained protection from major pathogens, including cytomegalovirus, adenovirus, BK virus, and Epstein-Barr virus in the absence of acute or chronic GVHD (96).

In an attempt to reprogram progenitor cells in order to evaluate their engraftment, differentiation, and safety, NSG mice CD34^+^ cells were ex-vivo transduced with a proprietary lentiviral vector encoding a human gene or a mock (GFP) vector. The result revealed that the mice treated with transduced CD34^+^ cells had lower aGVHD outcome such as lymphohistiocytic inflammatory cell infiltrates and microgranulomas in the liver and lungs in comparison to control mice injected with naive CD34^+^ cells (97).

## Cell Therapy of aGVHD

### Mesenchymal Stromal Cells

Mesenchymal stromal cells (MSCs) are non-hematopoietic self-renewal cells that have the ability of multipotent differentiation mainly into mesodermal lineages like chondrocytes, osteocytes and adipocytes (98–100). These cells that are known for their adherence capacity to plastic, neither express the hematopoietic and monocyte markers such as CD34, CD45 and CD14, nor endothelial markers like CD31 and CD144. Additionally, they do not express MHC II molecules like HLA-DR, and co-stimulation molecules like CD80 and CD86. However, they do express markers such as CD90, CD73, CD105, CD146, and CD29, plus a poor expression of MHC I molecules. MSCs can be isolated from different adult, prenatal and neonatal tissue sources including but not limited to BM, adipose tissue (AT), dental tissues, endometrium, amniotic fluid, umbilical cord and many others (101–103). It has been revealed that MSCs from diverse tissues have different regenerative and immunoregulatory features (101, 104). Moreover, source tissue diversities were correlated to variable expression quantities of highly procoagulant tissue factor (TF) CD142 on their cell surface (102), which remarkably affects their safety profile for intravenous (IV) infusion due to triggering of the “instant-blood-mediated inflammatory reaction” (IBMIR) (105). This is indeed a crucial aspect for the cells’ safety and efficacy profile (106) as also interestingly discussed by Moll G et al, for recent COVID-19 MSC based therapies (107).

MSCs can support hematopoietic cells and possess non-specific immunosuppressive and immunomodulatory functions against both innate and adaptive immune responses (108, 109). They can directly inhibit the proliferation of alloreactive T cells or convert them to Foxp3 expressing regulatory T cells through a cell-cell contact dependent and independent manner (**Figure 2**) (110–114). Additionally, MSCs can program macrophage plasticity by polarizing them towards less pro-inflammatory M1 and more anti-inflammatory M2 subpopulations (**Figure 2**) (115). Contrary to their in-vitro suppressive capacity when used in a 1:1 MSCs/T cells ratio, they had no clinical usefulness in terms of graft survival or severity of aGVHD in mice (116). However, few years ago Baron et al., revealed that, a third party, ex-vivo expanded, MSCs co-injection in a high risk, mismatched, unrelated-donor HSCT could reduce the severity of GVHD (117). On the other hand, co-injection of MSCs and HSCs in an HLA-identical sibling HSCT although resulted in a decrease of aGVHD severity, the incidence of relapse was remarkably higher (118). Recently a case report for a 15 years old boy, showed a dramatic decrease of aGVHD after treating with 2 × 10 (6) MSCs/kg 8 times in 4 weeks followed by MSCs administration once/week in the next 4 weeks (119).

Most cells release membrane-derived extracellular vesicles (EVs) carrying biomolecular payloads that offer significant potential in both detecting and treating diseases. EVs have a lipid bilayer and are ranging from 50 nm to ~2µm secreted from nearly all mammalian cell types (e.g., endothelial cells, neuronal cells, muscle cells, stem cells) that can be found in various body fluids such as breast milk, semen, saliva, urine, and serum (120). Based on their biogenesis pathways, EVs are categorized into three main classes: exosomes, microvesicles, and apoptotic bodies (121).

MSC-EVs could alter CD4^+^ T cells through an APC-related pathway, increasing the population of CD4^+^CD25^+^Foxp3^+^ Treg, consequently, increasing the immunosuppressive effects of MSC-EVs (**Figure 2**) (122). Furthermore, recent studies support the crucial role of MSC-EVs in regulating the M1/M2 macrophage subpopulation balance. For instance, MSC-EVs could interfere with the activation of M1 macrophages while favoring their M2 counterparts. This is accompanied by reduced secretion of TNFα, IFNγ, VEGF, and IL-12 and increased IL-10 production (123–125). EVs were shown to have the similar tissue repair capabilities as MSCs making them a promising non-cellular approach for GVHD treatment (126). It has been demonstrated that MSC-EVs could enhance the survival rate and reduce the grade of aGVHD in mouse models. This was followed by a modification in the naive and effector T cell ratio (127). Other studies reported reduced clinical symptoms including diarrhea and hormone consumption after MSC-EVs therapy. They showed that MSC-EV treatment reduced the PBMC secretion of IL-1β, TNFα, and IFNγ (128).

The encouraging point in using MSCs is that they are very well tolerated in-vivo, however, the efficiency of MSCs treatment is variable in different studies. This could be due to the fact that MSCs are very heterogeneous cells. Recently, we have demonstrated that compared to MSCs harvested from WT mice, their counterparts from TNFR2 KO mice are significantly disabled to suppress Teffs and convert them to Foxp3^+^Tregs (111). Sorting TNFR2 enriched MSCs or up-regulating this marker with a proper agonist could potentially lead to a more homogeneous cell product with increased immnuregulatory features. Taken together, the optimized source, dose, frequency and treatment intervals of MSCs administration require better understanding of the mechanisms of MSCs treatment.

As previously mentioned MSCs can exert their therapeutic effect either directly or indirectly through educating/reprogramming other cells such as macrophages and T cells. In the next sections, we discuss the role of regulatory macrophages and regulatory T cells in GVHD treatment.

### Regulatory Macrophages

Recipient macrophages are known to resist the conditioning regimen and to remain in patients for many weeks after HSCT (129). This might provide the opportunity to modulate donor T cell immunity. This hypothesis proved valid in a mouse model of GVHD indicating that macrophages resisted in lymphoid tissues after lethal irradiation and elimination by anti-colony stimulating factor 1 receptor (anti-CSF-1R), which is expressed on all monocytes and tissue macrophages and plays a key role in their homeostasis (130), led to exacerbated GVHD (131). They further showed that pre-transplant CSF-1 therapy could expand recipient regulatory macrophages resulting in amelioration of aGVHD through an IL-10 dependent mechanism. The infiltration of macrophages can add to GVHD occurrence, however, macrophages have different subpopulations which act differently in GVHD (132). Macrophages recruitment is one of the main steps in aGVHD initiation, and a higher ratio of M1/M2 macrophages correlates to a higher incidence of grades 2 to 4 acute GVHD (133, 134). Pro-inflammatory M1 macrophages have been shown to contribute and infiltrate more in aGVHD, whereas anti-inflammatory M2 macrophages are reported to be more predominant in cGVHD and refractory aGVHD. Due to the secretion of anti-inflammatory cytokines, such as IL-10 and TGFβ, M2 macrophages could suppress different immune cells, particularly T cells. Therefore, they could be potentially a good cell therapy product to target GVHD. Bouchlaka et al, showed that MSC educated M2 macrophages have enhanced CD206, CD163, IL-6, TGF-β, arginase-1 expression and reduced IL-12 and TNFα production and can attenuate GVHD. This was mostly due to controlled T cell proliferation and enhanced fibroblast proliferation (135). Very interestingly, it has been demonstrated that the polarization of M2 macrophages by MSCs is also TNF-TNFR2 dependent (136). This could demonstrate once more the importance of TNFR2 targeting to take the better advantage of M2 macrophages or change the balance of M1 and M2 macrophages in GVHD treatment.

### Regulatory T Cells

Natural regulatory T cells (nTregs) are defined as natural immunosuppressive cells that are able to inhibit alloreactive lymphocytes and control innate and adaptive immune responses (**Figure 2**) (137–140). Any impairment in Tregs functionality or imbalance in their recovery after HSCT is associated with a loss of tolerance and development of autoimmunity and also GVHD (141, 142). Compared to previous cell therapy approaches of aGVHD, Tregs are the most studied and applied cellular based therapy that has shown very promising results. Studies in mouse models have proved that depletion of Tregs before transplantation significantly accelerates the occurrence of aGVHD and inversely, others reported that adoptive transfer of freshly-purified donor Tregs or donor derived ex-vivo expanded Tregs were remarkably efficient to control aGVHD (143–145). The attractive point of Treg cell therapy is that GVL effect is acceptably preserved which is probably due to retention of donor T cells or differences in homing pattern of effector versus regulatory T cells (146). In addition to Treg suppressive activity they have other beneficial effects like facilitating the engraftment of hematopoietic cells and participating in immune reconstitution (60, 147). However, the low percentage of Tregs (5–10% of peripheral CD4^+^ T cells) represents a major obstacle for their vast clinical application. This barrier has been overcome by means of ex-vivo expansion of Tregs with anti-CD3 and anti-CD28 in the presence of IL-2, to yield non-specific polyclonal Tregs. Although, the application of polyclonal Tregs has shown promising outcomes in different complications such as GVHD (148), solid organ transplantations like kidney transplantation (149), non-immune diseases such as cardiovascular diseases, obesity, type 2 diabetes (T2D), and degenerative diseases (150), it was less convincing in other disorders such as type 1 diabetes (T1D) and multiple sclerosis (MS) mainly due to the heterogeneity of expanded Treg cell population (150, 151).

The other proposed solution was ex-vivo expansion of Tregs through TCR-mediated activation by alloantigen of recipient (recipient specific Treg or rs-Treg) in the presence of IL-2. This process permits to obtain a satisfying number of rs-Tregs that are capable of specifically suppressing donor T cells and consequently providing more promising results regarding aGVHD control compared with polyclonal Tregs (152, 153). These rs-Tregs could hamper the activation and differentiation of donor T cells in-vivo leading to a total and sustained protection of transplanted mice while preserving immune reconstitution and GVL effect (147, 154). Unfortunately, due to the difficulty to sort purified Tregs under clinical grade practice conditions, rs-Tregs involve a risk of contamination of cell product with highly alloreactive and thus pathogenic recipient specific effector T cells (rs-Teffs), which precludes their therapeutic application. To overcome this issue, Martin GH et al. suggested an alternative strategy utilizing Tregs which are specific for a single exogenous antigen (HY antigen specific Tregs or HY-Treg) that is neither expressed in donor nor in recipient (HY antigen is only expressed in males). In this case, the contaminating Teffs are maintained non-pathogenic as the exogenous antigen is transiently presented by few host APCs and is not expressed by host target organs of aGVHD. In a semi-allogeneic mouse model of HSCT, when both donors and recipients were female, the co-transfer of Teffs and HY-Tregs alone could not protect against aGVHD, however, modifying the gender of recipients to male mice that express HY antigen, was enough to completely protect against aGVHD. Alternatively, to re-activate HY-Tregs in-vivo in the presence of their cognate Ag, three intravenous injections of HY-peptide at D0, D3 and D6, resulted in entire protection against aGVHD (155). The hallmark of this strategy is that it potentially provides an OFF-ON system to benefit from the alloreactive effect of donor T cells on demand i.e. to destroy malignant cells when Tregs are off (non-activated), and to turn them on (activated with their cognate Ag) as soon as observing the primarily signs of aGVHD.

Further studies to identify the mechanism of action of Tregs in such an inflammatory environment revealed that in murine model of aGVHD, Treg immunosuppressive effect was dependent on the secretion of TNFα by Teffs and the expression of TNFR2 by Tregs. In this context, the blockade of TNFα-TNFR2 signaling pathway either by administration of an anti-TNFR2 mAb or harvesting Tregs from TNFR2-KO mice to block the possibility of signal transduction through the TNFR2, or using T cells harvested from TNFα KO mice led to the interruption in Treg suppressive function resulting in high grades of aGVHD (156, 157). The advantage of this finding is that it provides an OFF button for Tregs. Thus, after their proper immunosuppressive function (ON status) we have the possibility to turn them off until the next need.

Such promising results acquired with animal models over the last decade encouraged its application in human. Two phase 1 clinical trials using adoptive transfer or ex-vivo expanded Tregs before (day 4) or just after (day+1 +/− day+15) transplant resulted in notable reduction in the severity of aGVHD (158, 159). In further clinical update, Brunstein et al., have reported that the incidence of grades 2 to 4 aGVHD at 100 days was 9%, and cGVHD at 1 year was 0% without any difference in infection density (148). Moreover, a significant faster recovery of total CD4^+^T cells and a subset of naive CD4^+^T cells were observed. The rationale for using cord blood derived Tregs in the study by Brunstein et al. was in part based on their similar capacity to express the essential Tregs markers (160), in addition to their resistance against the classical immunosuppressant drugs that usually interfere with Treg viability or function, and therefore abrogate their therapeutic effect (161).

Another strategy to increase Treg percentage in patients is through in-vivo expansion of these cells by the administration of low doses of IL-2. Previous clinical studies had already revealed that IL-2 therapy induces the selective expansion of Tregs following HSCT and in patients with solid tumors (162–164). This strategy was tried in a phase 2 clinical trial which achieved an expansion of Tregs from a mean of 4.8% pre IL-2 to 11.1% after therapy, with the greatest change occurring in recipients of matched related donor transplants. Interestingly, no IL-2–treated patient developed grades 2 to 4 aGVHD. Additionally, IL-2–treated recipients maintained T cells reactive to viral and leukemia antigens and on the whole, the rate of infection was significantly lower compared with non-treated patients (165).

The low dose IL-2 administration was also studied in cGVHD with remarkable in-vivo Treg expansion and promising clinical results, particularly in pediatric patients (166–168).

### Innate Lymphoid Cells

Innate lymphoid cells (ILCs) are different from their B and T cell counterparts as they do not express rearranged Ag specific receptors (169). ILCs are a heterogeneous family of cells that are classified on the basis of their transcriptional factors and their functionality. Like T lymphocytes, ILCs are also grouped into cytotoxic and helper subsets. New classifications consider NK cells as cytotoxic ILCs that express T-bet and eomesodermin (Eomes) and are able to secrete IFNγ and TNFα, thus yielding cytotoxic effects (169, 170). Helper ILCs are further subdivided into three distinct populations: ILC1, ILC2 and ILC3. Briefly, ILC1 population needs T-bet for their development and they are able to secrete IFNγ. However, the difference between this population and NK cells is that they neither express Eomes nor exert cytotoxic activities (170). ILC2 express GATA3 and produce Th2 cytokines (171). Finally, ILC3 cells are themselves heterogeneous populations that are further subdivided into more subsets. They are known to express RORγt and to mainly produce IL-17 and IL-22 cytokines (172). In general, ILCs contribute to host defenses against a broad variety of pathogens (173, 174). In the context of GVHD, due to the damage caused by the conditioning regimen and the further tissue damage resulted from donor T cells attack, the role of ILCs is supposed to be essential. Hanash et al., identified intestinal ILC3 subset as the main IL-22–producing cells after TBI, highlighting their crucial role in the protection against epithelial cells damage and in preserving intestinal stem cells (175). The same results were reported by another team showing that IL-22 treatment in mice after HSCT could increase intestinal stem cell recovery, increase epithelial cell regeneration, and eventually reduce intestinal GVHD (176). The role of ILCs in tissue repair is not limited to intestinal cells since another study has described the promising role of ILC3 in thymic epithelial recovery, through IL-22 production, causing a more efficient T cell reconstitution (177). Similar results were obtained in lung epithelial tissue repair (178). The latter is in accordance with another study demonstrating a critical role of lung ILCs in restoring airway epithelial integrity and tissue homeostasis after infection with influenza virus (179). The possible protective effect of ILCs in aGVHD was firstly discussed by Hanash et al., showing that host-derived IL-22 could substantially limit aGVHD development (175). Moreover, Munneke et al., have suggested that once ILCs (regardless of origin, donor or recipient) are activated they could reduce aGVHD development and tissue damage (180). Nevertheless, the exact role of IL-22 in inflammatory conditions such as GVHD is not completely clear and might be controversial. For instance, Couturier et al, reported that the IL-22 deficiency in donor T cells could attenuate murine aGVHD mortality while preserving the GVL effect (181). Altogether, the positive role of ILCs in tissue repair, stabilization of stem cells and maintenance of tissue hemostasis is currently the subject of discussions and ILCs are potentially an interesting candidate to be tested in back to back therapies i.e. with classical pharmacological treatments or more interestingly with novel therapies such as gene therapies and regulatory T cells (182). In other words, testing the immune suppression caused by any of these approaches versus tissue repair and hemostasis that could be induced by ILCs.

### NKT Lymphocytes

NKT cells simultaneously express TCR and markers of NK cells. Within this population, invariant NKT (iNKT) are characterized by an invariant alpha chain of TCR that has a capacity to recognize glycolipids, like the *glycolipid* alpha-*galactosylceramide* (alpha-GalCer) antigen presented by CD1d molecules (183, 184). This glycolipid induces a fast and massive activation of NKT cells which are involved in the regulation of allogeneic responses *via* production of IL-4 and IFNγ. They can also regulate other cells of the immune system towards a tolerogenic or a cytotoxic response, particularly against tumors (185–187). In a mouse model, it was shown that CD4^-^CD8^-^ iNKT lymphocytes of bone marrow origin, could control aGVHD without attenuation of GVL effect (188). Authors also suggested that this protection effect is through production of IL-4 by NKT cells that can consecutively induce Treg proliferation. In another study, administering a low dose of CD4^+^NKT at the same time of the BM graft significantly reduced the incidence of aGVHD. Once again, this was associated with IL-4 secretion by NKT cells and subsequently altering the secretion of pro-inflammatory cytokines such as IFNγ and TNFα by donor T cells, without hampering their proliferation (189). In patients who received Total Lymphoid Irradiation (TLI) conditioning regimen, a very good reconstitution of iNKT was observed and this was linked to a remarkable decrease in the incidence of higher grades of aGVHD (190). Moreover, Rubio et al., have provided a proof of concept that early post-allogeneic HSCT iNKT cell recovery can predict the occurrence of aGVHD and an improved overall survival (191). This was confirmed in another study showing that proportion of CD4^-^ iNKT cells of the graft could be predictive of aGVHD in recipients (192).

### Endothelial Progenitor Cells

Endothelial progenitor cells (EPCs) are the BM-derived hematopoietic cells that are responsible for neo-vascularization and repairing tissue damages at the endothelial sites (193). These cells that express classical endothelial markers such CD31, CD144, VEGFR2 and CD133 demonstrate some unique features that make them especially interesting for treatment of degenerative, cardiovascular and hematopoietic disorder. For instance, Loisel et al. have shown a successful administration of autologous EPCs for the treatment of right ventricle (RV) failure in a piglet model of chronic thromboembolic pulmonary hypertension (CTEPH) (194). Similar to MSCs, EPCs have shown some levels of immunosuppressive and immunomodulatory properties (195). Our team has recently demonstrated that human EPC derived from CB are tolerated in xenogeneic mouse models of ischemia and contributed to vascular formation (196). We further revealed that EPCs’ immunosuppressive effect was entirely TNFR2 dependent since administration of an anti-TNFR2 mAb abolished their regulatory functions (197). Accordingly, we showed that priming EPCs with TNFα enhances their immunosuppressive effect through a TNFR2 dependent interaction (198). These interesting features encouraged scientists to evaluate their therapeutic effect in GVHD models. EPCs injection was reported to be have some protective roles in accelerating hematopoietic and immune reconstitution, restoring vascular niche in BM and ameliorating GVHD grade through improving the integrity of BM sinusoidal endothelial cells (199–202). Further investigations revealed that the administration of anti-vascular endothelial cadherin antibody **(AAVE**) remarkably interrupted those mentioned effects (200). Based on our recent experiences, we think it would be very interesting to specifically target TNFR2 molecule in EPCs *via* its proper agonist, in order to selectively upregulate this marker and benefit from increased EPC immunosuppressive and pro-angiogenic effects. Controlling these two crucial aspects leads to higher HSCs engraftment, better immune reconstitution and, if necessary, improved GVHD prevention.

## Conclusions

In spite of great advancements in treating GVHD, it still remains a major complication of HSCT. Here, we described a series of novel therapeutic approaches that target different cells that contribute to GVHD occurrence. Additionally, we have discussed the application and the potential therapeutic benefits of a variety of cells with immunoregulatory functions with the special attention in Tregs that have been proved to be a very promising approach to control GVHD. Cell free therapies including the administration of EVs, in-vivo amplification of regulatory cells and targeting immune checkpoint signaling pathways such as the TNF-TNFR2 axis are among some new emerging approaches to selectively control the reaction and intensity of the immune response which potentially could lead to better control of GVHD.

## Author Contributions

SN, ML, and SS wrote the manuscript. SN, ML, and GU reviewed and revised the manuscript. All authors contributed to the article and approved the submitted version.

## Funding

SN was supported by a governmental grant *via* ‘‘l’Agence Nationale de la Recherche’’ in the form of ‘‘programme d’Investissements d’avenir’’ with the grant number: ANR_15-RHUS60002.

## Conflict of Interest

SN is the CEO of CellMedEx Company.

The remaining authors declare that the research was conducted in the absence of any commercial or financial relationships that could be construed as a potential conflict of interest.

## Abbreviations

ATG, anti-thymocyte globulin; BM, bone marrow; CD, cluster of differentiation; EVs, extracellular vesicles; GVHD, graft versus host disease; GVL, graft versus leukemia; GVT, graft versus tumor; HSCs, hematopoietic stem cells; HSCT, hematopoietic stem cell transplantation; IL, interleukin; ILC, innate lymphoid cells; MSCs, mesenchymal stromal cells; NK, natural killer cells; PB, peripheral blood; TBI, total body irradiation; Tc, cytotoxic T cells; TCR, T cell receptor; Teffs, effector T cells; Th, T helper cells; TNFR2, tumor necrosis factor receptor 2; TNFα, tumor nerosis factor alpha; Treg, regulatory T cells; UCB, umbilical cord blood.

## References

[1] Thomas JW Plenderleith IH Clements DV Landi S Observations in immunotherapy of lymphoma and melanoma patients. Clin Exp Immunol (1975) 21(1):82–96. 1102163 PMC1538246

[2] Shenoy S Hematopoietic stem-cell transplantation for sickle cell disease: current evidence and opinions. Ther Adv Hematol (2013) 4(5):335–44. 10.1177/2040620713483063 PMC3766347 24082994

[3] Dietz AC Lucchini G Samarasinghe S Pulsipher MA Evolving hematopoietic stem cell transplantation strategies in severe aplastic anemia. Curr Opin Pediatr (2016) 28(1):3–11. 10.1097/MOP.0000000000000299 26626557 PMC4725196

[4] Martin PJ Hansen JA Buckner CD Sanders JE Deeg HJ Stewart P Effects of in vitro depletion of T cells in HLA-identical allogeneic marrow grafts. Blood (1985) 66(3):664–72. 10.1182/blood.V66.3.664.664 3896348

[5] Mackall CL Gress RE Pathways of T-cell regeneration in mice and humans: implications for bone marrow transplantation and immunotherapy. Immunol Rev (1997) 157:61–72. 10.1111/j.1600-065X.1997.tb00974.x 9255622

[6] Horowitz MM Gale RP Sondel PM Goldman JM Kersey J Kolb HJ Graft-versus-leukemia reactions after bone marrow transplantation. Blood (1990) 75(3):555–62. 10.1182/blood.V75.3.555.bloodjournal753555 2297567

[7] Barnes DW Loutit JF Treatment of murine leukaemia with x-rays and homologous bone marrow. II. Br J Haematol (1957) 3(3):241–52. 10.1111/j.1365-2141.1957.tb05793.x 13460193

[8] Butturini A Gale RP The role of T-cells in preventing relapse in chronic myelogenous leukemia. Bone Marrow Transplant (1987) 2(4):351–4. 3332182

[9] Truitt RL Ash RC Manipulation of T-cell content in transplanted human bone marrow: effect on GVH and GVL reactions. Prog Clin Biol Res (1987) 244:409–21. 3310003

[10] Kärre K Ljunggren HG Piontek G Kiessling R Selective rejection of H-2-deficient lymphoma variants suggests alternative immune defence strategy. Nature (1986) 319(6055):675–8. 10.1038/319675a0 3951539

[11] Ljunggren HG Kärre K In search of the “missing self”: MHC molecules and NK cell recognition. Immunol Today (1990) 11(7):237–44. 10.1016/0167-5699(90)90097-s 2201309

[12] Caligiuri MA Human natural killer cells. Blood (2008) 112(3):461–9. 10.1182/blood-2007-09-077438 PMC2481557 18650461

[13] Ferrara JLM Levine JE Reddy P Holler E Graft-versus-host disease. Lancet (2009) 373(9674):1550–61. 10.1016/S0140-6736(09)60237-3 PMC2735047 19282026

[14] Barnes DW Loutit JF Micklem HS “Secondary disease” of radiation chimeras: a syndrome due to lymphoid aplasia. Ann N Y Acad Sci (1962) 99:374–85. 10.1111/j.1749-6632.1962.tb45321.x 13969359

[15] Billingham RE The biology of graft-versus-host reactions. Harvey Lect. (1966) 62:21–78. 4875305

[16] Kernan NA Collins NH Juliano L Cartagena T Dupont B O’Reilly RJ Clonable T lymphocytes in T cell-depleted bone marrow transplants correlate with development of graft-v-host disease. Blood (1986) 68(3):770–3. 10.1182/blood.V68.3.770.bloodjournal683770 3527302

[17] Anasetti C Beatty PG Storb R Martin PJ Mori M Sanders JE Effect of HLA incompatibility on graft-versus-host disease, relapse, and survival after marrow transplantation for patients with leukemia or lymphoma. Hum Immunol (1990) 29(2):79–91. 10.1016/0198-8859(90)90071-V 2249952

[18] Turpeinen H Ojala PJ Ojala K Miettinen M Volin L Partanen J Minor histocompatibility antigens as determinants for graft-versus-host disease after allogeneic haematopoietic stem cell transplantation. Int J Immunogenet (2013) 40(6):495–501. 10.1111/iji.12051 23480177

[19] Waterhouse M GVHD meets GWAS. Blood (2015) 126(25):2662–3. 10.1182/blood-2015-10-674093 26679545

[20] Sackstein R A revision of Billingham’s tenets: the central role of lymphocyte migration in acute graft-versus-host disease. Biol Blood Marrow Transplant (2006) 12(1 Suppl 1):2–8. 10.1016/j.bbmt.2005.09.015 16399577

[21] Blazar BR Murphy WJ Bone marrow transplantation and approaches to avoid graft-versus-host disease (GVHD). Philos Trans R Soc Lond B Biol Sci (2005) 360(1461):1747–67. 10.1098/rstb.2005.1701 PMC1569546 16147539

[22] Elfeki MA Pungpapong S Genco PV Nakhleh RE Nguyen JH Harnois DM Graft-versus-host disease after orthotopic liver transplantation: multivariate analysis of risk factors. Clin Transplant (2015) 29(12):1063–6. 10.1111/ctr.12627 26358521

[23] Morisse-Pradier H Nove-Josserand R Philit F Senechal A Berger F Callet-Bauchu E [Graft-versus-host disease, a rare complication of lung transplantation]. Rev Pneumol Clin (2016) 72(1):101–7. 10.1016/j.pneumo.2015.05.004 26209034

[24] Kopolovic I Ostro J Tsubota H Lin Y Cserti-Gazdewich CM Messner HA A systematic review of transfusion-associated graft-versus-host disease. Blood (2015) 126(3):406–14. 10.1182/blood-2015-01-620872 25931584

[25] Anasetti C Martin PJ Hansen JA Lin Y Cserti-Gazdewich CM Messner HA A phase I-II study evaluating the murine anti-IL-2 receptor antibody 2A3 for treatment of acute graft-versus-host disease. Transplantation (1990) 50(1):49–54. 10.1097/00007890-199007000-00010 2368150

[26] Sullivan KM Agura E Anasetti C Appelbaum F Badger C Bearman S Chronic graft-versus-host disease and other late complications of bone marrow transplantation. Semin Hematol (1991) 28(3):250–9. 1887253

[27] Sullivan KM Mori M Sanders J Siadak M Witherspoon RP Anasetti C Late complications of allogeneic and autologous marrow transplantation. Bone Marrow Transplant (1992) 10 Suppl 1:127–34. 1521083

[28] Ghimire S Weber D Mavin E Wang XN Dickinson AM Holler E Pathophysiology of GvHD and Other HSCT-Related Major Complications. Front Immunol (2017) 8:79:79. 10.3389/fimmu.2017.00079 28373870 PMC5357769

[29] Blazar BR Murphy WJ Abedi M Advances in graft-versus-host disease biology and therapy. Nat Rev Immunol (2012) 12(6):443–58. 10.1038/nri3212 PMC3552454 22576252

[30] Bram RJ Hung DT Martin PK Schreiber SL Crabtree GR Identification of the immunophilins capable of mediating inhibition of signal transduction by cyclosporin A and FK506: roles of calcineurin binding and cellular location. Mol Cell Biol (1993) 13(8):4760–9. 10.1128/MCB.13.8.4760 PMC360102 7687744

[31] Clipstone NA Crabtree GR Identification of calcineurin as a key signalling enzyme in T-lymphocyte activation. Nature (1992) 357(6380):695–7. 10.1038/357695a0 1377362

[32] Powles RL Clink HM Spence D Morgenstern G Watson JG Selby PJ Cyclosporin A to prevent graft-versus-host disease in man after allogeneic bone-marrow transplantation. Lancet (1980) 1(8164):327–9. 10.1016/S0140-6736(80)90881-8 6101787

[33] Storb R Deeg HJ Farewell V Doney K Appelbaum F Beatt P Marrow transplantation for severe aplastic anemia: methotrexate alone compared with a combination of methotrexate and cyclosporine for prevention of acute graft-versus-host disease. Blood (1986) 68(1):119–25. 10.1182/blood.V68.1.119.bloodjournal681119 3521761

[34] Finke J Bethge WA Schmoor C Ottinger HD Stelljes M Zander AR Standard graft-versus-host disease prophylaxis with or without anti-T-cell globulin in haematopoietic cell transplantation from matched unrelated donors: a randomised, open-label, multicentre phase 3 trial. Lancet Oncol (2009) 10(9):855–64. 10.1016/S1470-2045(09)70225-6 19695955

[35] Juric MK Ghimire S Ogonek J Weissinger EM Holler E van Rood JJ Milestones of Hematopoietic Stem Cell Transplantation - From First Human Studies to Current Developments. Front Immunol (2016) 7:470:470. 10.3389/fimmu.2016.00470 27881982 PMC5101209

[36] Slavin S Nagler A Naparstek E Kapelushnik Y Aker M Cividalli G Nonmyeloablative stem cell transplantation and cell therapy as an alternative to conventional bone marrow transplantation with lethal cytoreduction for the treatment of malignant and nonmalignant hematologic diseases. Blood (1998) 91(3):756–63. 10.1182/blood.V91.3.756 9446633

[37] Brissot E Chevallier P Guillaume T Delaunay J Ayari S Dubruille V Prophylaxis with mycophenolate mofetil and CsA can decrease the incidence of severe acute GVHD after antithymocyte globulin-based reduced-intensity preparative regimen and allo-SCT from HLA-matched unrelated donors. Bone Marrow Transplant (2010) 45(4):786–8. 10.1038/bmt.2009.218 19718059

[38] Koreth J Antin JH Current and future approaches for control of graft-versus-host disease. Expert Rev Hematol (2008) 1(1):111. 10.1586/17474086.1.1.111 20151032 PMC2819425

[39] Pilon CB Petillon S Naserian S Martin GH Badoual C Lang P Administration of low doses of IL-2 combined to rapamycin promotes allogeneic skin graft survival in mice. Am J Transplant (2014) 14(12):2874–82. 10.1111/ajt.12944 25394722

[40] Hamdani S Thiolat A Naserian S Grondin C Moutereau S Hulin A Delayed and short course of rapamycin prevents organ rejection after allogeneic liver transplantation in rats. World J Gastroenterol (2017) 23(38):6962–72. 10.3748/wjg.v23.i38.6962 PMC5658314 29097869

[41] Battaglia M Stabilini A Migliavacca B Horejs-Hoeck J Kaupper T Roncarolo M-G Rapamycin promotes expansion of functional CD4+CD25+FOXP3+ regulatory T cells of both healthy subjects and type 1 diabetic patients. J Immunol (2006) 177(12):8338–47. 10.4049/jimmunol.177.12.8338 17142730

[42] Yang F Li Y Zhang Q Tan L Peng L Zhao Y The Effect of Immunosuppressive Drugs on MDSCs in Transplantation. J Immunol Res (2018) 2018:5414808. 10.1155/2018/5414808 30057917 PMC6051033

[43] Cutler C Antin JH Sirolimus immunosuppression for graft-versus-host disease prophylaxis and therapy: an update. Curr Opin Hematol (2010) 17(6):500–4. 10.1097/MOH.0b013e32833e5b2e 20717025

[44] Johnston L Florek M Armstrong R McCune JS Arai S Brown J Sirolimus and mycophenolate mofetil as GVHD prophylaxis in myeloablative, matched-related donor hematopoietic cell transplantation. Bone Marrow Transplant (2012) 47(4):581–8. 10.1038/bmt.2011.104 PMC3163055 21552302

[45] Sandmaier BM Kornblit B Storer BE Olesen G Maris MB Langston AA Addition of sirolimus to standard cyclosporine plus mycophenolate mofetil-based graft-versus-host disease prophylaxis for patients after unrelated non-myeloablative haemopoietic stem cell transplantation: a multicentre, randomised, phase 3 trial. Lancet Haematol (2019) 6(8):e409–18. 10.1016/S2352-3026(19)30088-2 PMC6686903 31248843

[46] Haase D Starke M Puan KJ Lai TS Rotzschke O Immune modulation of inflammatory conditions: regulatory T cells for treatment of GvHD. Immunol Res (2012) 53(1-3):200–12. 10.1007/s12026-012-8267-9 22418725

[47] Martin PJ Rizzo JD Wingard JR Ballen K Curtin PT Cutler C First- and second-line systemic treatment of acute graft-versus-host disease: recommendations of the American Society of Blood and Marrow Transplantation. Biol Blood Marrow Transplant (2012) 18(8):1150–63. 10.1016/j.bbmt.2012.04.005 PMC3404151 22510384

[48] Van Lint MT Uderzo C Locasciulli A Majolino I Scimé R Locatelli F Early treatment of acute graft-versus-host disease with high- or low-dose 6-methylprednisolone: a multicenter randomized trial from the Italian Group for Bone Marrow Transplantation. Blood (1998) 92(7):2288–93. 9746766

[49] Alousi AM Weisdorf DJ Logan BR Bolaños-Meade J Carter S Difronzo N Etanercept, mycophenolate, denileukin, or pentostatin plus corticosteroids for acute graft-versus-host disease: a randomized phase 2 trial from the Blood and Marrow Transplant Clinical Trials Network. Blood (2009) 114(3):511–7. 10.1182/blood-2009-03-212290 PMC2713466 19443659

[50] Bolaños-Meade J Logan BR Alousi AM Antin JH Barowski K Carter SL Phase 3 clinical trial of steroids/mycophenolate mofetil vs steroids/placebo as therapy for acute GVHD: BMT CTN 0802. Blood (2014) 124(22):3221–7. 10.1182/blood-2014-06-577023. quiz 3335. PMC4239331 25170121

[51] Zeiser R von Bubnoff N Butler J Mohty M Niederwieser D Or R Ruxolitinib for Glucocorticoid-Refractory Acute Graft-versus-Host Disease. N Engl J Med (2020) 382(19):1800–10. 10.1056/NEJMoa1917635 32320566

[52] Choi J Cooper ML Alahmari B Ritchey J Collins L Holt M Pharmacologic blockade of JAK1/JAK2 reduces GvHD and preserves the graft-versus-leukemia effect. PloS One (2014) 9(10):e109799. 10.1371/journal.pone.0109799 25289677 PMC4188578

[53] Nicholson SE Oates AC Harpur AG Ziemiecki A Wilks AF Layton JE Tyrosine kinase JAK1 is associated with the granulocyte-colony-stimulating factor receptor and both become tyrosine-phosphorylated after receptor activation. Proc Natl Acad Sci U S A (1994) 91(8):2985–8. 10.1073/pnas.91.8.2985 PMC43499 7512720

[54] Heine A Held SAE Daecke SN Wallner S Yajnanarayana SP Kurts C The JAK-inhibitor ruxolitinib impairs dendritic cell function in vitro and in vivo. Blood (2013) 122(7):1192–202. 10.1182/blood-2013-03-484642 23770777

[55] Spoerl S Mathew NR Bscheider M Schmitt-Graeff A Chen S Mueller T Activity of therapeutic JAK 1/2 blockade in graft-versus-host disease. Blood (2014) 123(24):3832–42. 10.1182/blood-2013-12-543736 24711661

[56] Chao NJ Chen BJ Prophylaxis and treatment of acute graft-versus-host disease. Semin Hematol (2006) 43(1):32–41. 10.1053/j.seminhematol.2005.09.007 16412787

[57] Ferrara JLM Yanik G Acute graft versus host disease: pathophysiology, risk factors, and prevention strategies. Clin Adv Hematol Oncol (2005) 3(5):415–9. 428. 16167015

[58] Vargas-Díez E García-Díez A Marín A Fernández-Herrera J Life-threatening graft-vs-host disease. Clin Dermatol (2005) 23(3):285–300. 10.1016/j.clindermatol.2004.06.005 15896544

[59] Kernan NA Bordignon C Keever CA Cunningham I Castro-Malaspina H Collins NH Graft failures after T cell depleted marrow transplants for leukemia: clinical and in vitro characteristics. Transplant Proc (1987) 19(6 Suppl 7):29–32. 2962353

[60] Hanash AM Levy RB Donor CD4+CD25+ T cells promote engraftment and tolerance following MHC-mismatched hematopoietic cell transplantation. Blood (2005) 105(4):1828–36. 10.1182/blood-2004-08-3213 15494429

[61] Aversa F Tabilio A Velardi A Cunningham I Terenzi A Falzetti F Treatment of high-risk acute leukemia with T-cell-depleted stem cells from related donors with one fully mismatched HLA haplotype. N Engl J Med (1998) 339(17):1186–93. 10.1056/NEJM199810223391702 9780338

[62] Bethge WA Faul C Bornhäuser M Stuhler G Beelen DW Lang P Haploidentical allogeneic hematopoietic cell transplantation in adults using CD3/CD19 depletion and reduced intensity conditioning: an update. Blood Cells Mol Dis (2008) 40(1):13–9. 10.1016/j.bcmd.2007.07.001 17869547

[63] Bertaina A Merli P Rutella S Pagliara D Bernardo ME Masetti R HLA-haploidentical stem cell transplantation after removal of αβ+ T and B cells in children with nonmalignant disorders. Blood (2014) 124(5):822–6. 10.1182/blood-2014-03-563817 24869942

[64] Ciceri F Labopin M Aversa F Rowe JM Bunjes D Lewalle P A survey of fully haploidentical hematopoietic stem cell transplantation in adults with high-risk acute leukemia: a risk factor analysis of outcomes for patients in remission at transplantation. Blood (2008) 112(9):3574–81. 10.1182/blood-2008-02-140095 18606875

[65] Tamari R Chung SS Papadopoulos EB Jakubowski AA Hilden P Devlin SM CD34-Selected Hematopoietic Stem Cell Transplants Conditioned with Myeloablative Regimens and Antithymocyte Globulin for Advanced Myelodysplastic Syndrome: Limited Graft-versus-Host Disease without Increased Relapse. Biol Blood Marrow Transplant (2015) 21(12):2106–14. 10.1016/j.bbmt.2015.07.010 PMC4764129 26187863

[66] Barba P Hilden P Devlin SM Maloy M Dierov D Nieves J Ex Vivo CD34+-Selected T Cell-Depleted Peripheral Blood Stem Cell Grafts for Allogeneic Hematopoietic Stem Cell Transplantation in Acute Leukemia and Myelodysplastic Syndrome Is Associated with Low Incidence of Acute and Chronic Graft-versus-Host Disease and High Treatment Response. Biol Blood Marrow Transplant (2017) 23(3):452–8. 10.1016/j.bbmt.2016.12.633 PMC5398850 28017734

[67] Ramsay NK Kersey JH Robison LL McGlave PB Woods WG Krivit W A randomized study of the prevention of acute graft-versus-host disease. N Engl J Med (1982) 306(7):392–7. 10.1056/NEJM198202183060703 7035950

[68] Russell JA Turner AR Larratt L Chaudhry A Morris D Brown C Adult recipients of matched related donor blood cell transplants given myeloablative regimens including pretransplant antithymocyte globulin have lower mortality related to graft-versus-host disease: a matched pair analysis. Biol Blood Marrow Transplant (2007) 13(3):299–306. 10.1016/j.bbmt.2006.10.017 17317583

[69] Bacigalupo A Lamparelli T Bruzzi P Guidi S Alessandrino PE di Bartolomeo P Antithymocyte globulin for graft-versus-host disease prophylaxis in transplants from unrelated donors: 2 randomized studies from Gruppo Italiano Trapianti Midollo Osseo (GITMO). Blood (2001) 98(10):2942–7. 10.1182/blood.V98.10.2942 11698275

[70] Bacigalupo A Lamparelli T Barisione G Bruzzi P Guidi S Alessandrino PE Thymoglobulin prevents chronic graft-versus-host disease, chronic lung dysfunction, and late transplant-related mortality: long-term follow-up of a randomized trial in patients undergoing unrelated donor transplantation. Biol Blood Marrow Transplant (2006) 12(5):560–5. 10.1016/j.bbmt.2005.12.034 16635791

[71] Bonifazi F Rubio M-T Bacigalupo A Boelens JJ Finke J Greinix H Rabbit ATG/ATLG in preventing graft-versus-host disease after allogeneic stem cell transplantation: consensus-based recommendations by an international expert panel. Bone Marrow Transplant (2020) 55(6):1093–102. 10.1038/s41409-020-0792-x PMC7269907 31969678

[72] Waid TH Thompson JS Siemionow M Brown SA T10B9 monoclonal antibody: a short-acting nonstimulating monoclonal antibody that spares gammadelta T-cells and treats and prevents cellular rejection. Drug Des Devel Ther (2009) 3:205–12. 10.2147/DDDT.S2750 PMC2769243 19920935

[73] Wagner JE Thompson JS Carter SL Kernan NA Unrelated Donor Marrow Transplantation Trial. Effect of graft-versus-host disease prophylaxis on 3-year disease-free survival in recipients of unrelated donor bone marrow (T-cell Depletion Trial): a multi-centre, randomised phase II-III trial. Lancet (2005) 366(9487):733–41. 10.1016/S0140-6736(05)66996-6 16125590

[74] Gilleece MH Dexter TM Effect of Campath-1H antibody on human hematopoietic progenitors in vitro. Blood (1993) 82(3):807–12. 10.1182/blood.V82.3.807.807 7687895

[75] Coles AJ Wing MG Molyneux P Paolillo A Davie CM Hale G Monoclonal antibody treatment exposes three mechanisms underlying the clinical course of multiple sclerosis. Ann Neurol (1999) 46(3):296–304. 10.1002/1531-8249(199909)46:3<296::AID-ANA4>3.0.CO;2-# 10482259

[76] Finazzi MC Boschini C Craddock C Rambaldi A Ward J Malladi RK Characteristics of graft-versus-host disease occurring after alemtuzumab-containing allogeneic stem cell transplants: incidence, organ involvement, risk factors and survival. Br J Haematol (2020) 188(4):550–9. 10.1111/bjh.16200 31713861

[77] Kanda J Lopez RD Rizzieri DA Alemtuzumab for the prevention and treatment of graft-versus-host disease. Int J Hematol (2011) 93(5):586–93. 10.1007/s12185-011-0802-2 21369856

[78] Luznik L O’Donnell PV Symons HJ Chen AR Leffell MS Zahurak M HLA-haploidentical bone marrow transplantation for hematologic malignancies using nonmyeloablative conditioning and high-dose, posttransplantation cyclophosphamide. Biol Blood Marrow Transplant (2008) 14(6):641–50. 10.1016/j.bbmt.2008.03.005 PMC2633246 18489989

[79] Ganguly S Ross DB Panoskaltsis-Mortari A Kanakry CG Blazar BR Levy RB Donor CD4+ Foxp3+ regulatory T cells are necessary for posttransplantation cyclophosphamide-mediated protection against GVHD in mice. Blood (2014) 124(13):2131–41. 10.1182/blood-2013-10-525873 PMC4186542 25139358

[80] Luznik L Bolaños-Meade J Zahurak M Chen AR Smith BD Brodsky R High-dose cyclophosphamide as single-agent, short-course prophylaxis of graft-versus-host disease. Blood (2010) 115(16):3224–30. 10.1182/blood-2009-11-251595 PMC2858487 20124511

[81] Reshef R Luger SM Hexner EO Loren AW Frey NV Nasta SD Blockade of lymphocyte chemotaxis in visceral graft-versus-host disease. N Engl J Med (2012) 367(2):135–45. 10.1056/NEJMoa1201248 PMC3568501 22784116

[82] Moy RH Huffman AP Richman LP Crisalli L Wang XK Hoxie JA Clinical and immunologic impact of CCR5 blockade in graft-versus-host disease prophylaxis. Blood (2017) 129(7):906–16. 10.1182/blood-2016-08-735076 PMC5314813 28057639

[83] Ryu J Jhun J Park M-J Baek J-A Kim S-Y Cho K-H FTY720 ameliorates GvHD by blocking T lymphocyte migration to target organs and by skin fibrosis inhibition. J Transl Med (2020) 18(1):225. 10.1186/s12967-020-02386-w 32505218 PMC7276082

[84] Huu DL Matsushita T Jin G Hamaguchi Y Hasegawa M Takehara K FTY720 ameliorates murine sclerodermatous chronic graft-versus-host disease by promoting expansion of splenic regulatory cells and inhibiting immune cell infiltration into skin. Arthritis Rheumatol (2013) 65(6):1624–35. 10.1002/art.37933 23508350

[85] Gauthier J Vermersch P Chauvet P Varlet P Coiteux V Magro L Successful treatment with fingolimod of graft-versus-host disease of the central nervous system. Blood Adv (2018) 2(1):10–3. 10.1182/bloodadvances.2017011478 PMC5761625 29344580

[86] Kekre N Kim HT Hofer J Ho VT Koreth J Armand P Phase II trial of natalizumab with corticosteroids as initial treatment of gastrointestinal acute graft-versus-host disease. Bone Marrow Transplant (2020). 10.1038/s41409-020-01049-0 32895491

[87] Furman PA McGuirt PV Keller PM Fyfe JA Elion GB Inhibition by acyclovir of cell growth and DNA synthesis of cells biochemically transformed with herpesvirus genetic information. Virology (1980) 102(2):420–30. 10.1016/0042-6822(80)90109-9 6245517

[88] Davidson RL Kaufman ER Crumpacker CS Schnipper LE Inhibition of herpes simplex virus transformed and nontransformed cells by acycloguanosine: mechanisms of uptake and toxicity. Virology (1981) 113(1):9–19. 10.1016/0042-6822(81)90132-X 6267792

[89] Springer CJ Niculescu-Duvaz I Prodrug-activating systems in suicide gene therapy. J Clin Invest (2000) 105(9):1161–7. 10.1172/JCI10001 PMC315452 10791987

[90] Bondanza A Valtolina V Magnani Z Ponzoni M Fleischhauer K Bonyhadi M Suicide gene therapy of graft-versus-host disease induced by central memory human T lymphocytes. Blood (2006) 107(5):1828–36. 10.1182/blood-2005-09-3716 16293601

[91] Cohen JL Boyer O Salomon B Onclercq R Charlotte F Bruel S Prevention of graft-versus-host disease in mice using a suicide gene expressed in T lymphocytes. Blood (1997) 89(12):4636–45. 10.1182/blood.V89.12.4636 9192790

[92] Cohen JL Boyer O Salomon B Onclerco R Depetris D Lejeune L Fertile homozygous transgenic mice expressing a functional truncated herpes simplex thymidine kinase delta TK gene. Transgenic Res (1998) 7(5):321–30. 10.1023/a:1008893206208 9859221

[93] Mailly L Leboeuf C Tiberghien P Baumert T Robinet E Genetically engineered T-cells expressing a ganciclovir-sensitive HSV-tk suicide gene for the prevention of GvHD. Curr Opin Invest Drugs (2010) 11(5):559–70. 20419602

[94] Maury S Rosenzwajg M Redjoul R Marcais A Xhaard A Cherai M Lymphodepletion followed by infusion of suicide gene-transduced donor lymphocytes to safely enhance their antitumor effect: a phase I/II study. Leukemia (2014) 28(12):2406–10. 10.1038/leu.2014.237 25102947

[95] Cohen JL Boyer O Klatzmann D Would suicide gene therapy solve the “T-cell dilemma” of allogeneic bone marrow transplantation? Immunol Today (1999) 20(4):172–6. 10.1016/S0167-5699(98)01420-0 10203714

[96] Zhou X Di Stasi A Tey S-K Krance RA Martinez C Leung KS Long-term outcome after haploidentical stem cell transplant and infusion of T cells expressing the inducible caspase 9 safety transgene. Blood (2014) 123(25):3895–905. 10.1182/blood-2014-01-551671 PMC4064331 24753538

[97] Chandra S Cristofori P Fonck C O’Neill CA Ex Vivo Gene Therapy: Graft-versus-host Disease (GVHD) in NSG^TM^ (NOD.Cg-Prkdcscid Il2rgtm1Wjl/SzJ) Mice Transplanted with CD34+ Human Hematopoietic Stem Cells. Toxicol Pathol (2019) 47(5):656–60. 10.1177/0192623319844484 31064282

[98] Pittenger MF Mackay AM Beck SC Jaiswal RK Douglas R Mosca JD Multilineage potential of adult human mesenchymal stem cells. Science (1999) 284(5411):143–7. 10.1126/science.284.5411.143 10102814

[99] Sanchez-Ramos J Song S Cardozo-Pelaez F Hazzi C Stedeford T Willing A Adult bone marrow stromal cells differentiate into neural cells in vitro. Exp Neurol (2000) 164(2):247–56. 10.1006/exnr.2000.7389 10915564

[100] Toma C Pittenger MF Cahill KS Byrne BJ Kessler PD Human mesenchymal stem cells differentiate to a cardiomyocyte phenotype in the adult murine heart. Circulation (2002) 105(1):93–8. 10.1161/hc0102.101442 11772882

[101] Hass R Kasper C Böhm S Jacobs R Different populations and sources of human mesenchymal stem cells (MSC): A comparison of adult and neonatal tissue-derived MSC. Cell Commun Signal (2011) 9:12. 10.1186/1478-811X-9-12 21569606 PMC3117820

[102] Moll G Ankrum JA Kamhieh-Milz J Bieback K Ringdén O Volk H-D Intravascular Mesenchymal Stromal/Stem Cell Therapy Product Diversification: Time for New Clinical Guidelines. Trends Mol Med (2019) 25(2):149–63. 10.1016/j.molmed.2018.12.006 30711482

[103] Chinnici CM Pietrosi G Iannolo G Amico G Cuscino N Pagano V Mesenchymal stromal cells isolated from human fetal liver release soluble factors with a potential role in liver tissue repair. Differentiation (2019) 105:14–26. 10.1016/j.diff.2018.12.001 30553176

[104] Yu Y Valderrama AV Han Z Uzan G Naserian S Oberlin E Human fetal liver MSCs are more effective than adult bone marrow MSCs for their immunosuppressive, immunomodulatory and Foxp3+ T regs induction capacity. Research Square [PREPRINT] (Version 2) (2020). 10.21203/rs.3.rs-40561/v1. PMC7888159 33597011

[105] Moll G Rasmusson-Duprez I von Bahr L Connolly-Andersen A-M Elgue G Funke L Are therapeutic human mesenchymal stromal cells compatible with human blood? Stem Cells (2012) 30(7):1565–74. 10.1002/stem.1111 22522999

[106] Caplan H Olson SD Kumar A George M Prabhakara KS Wenzel P Mesenchymal Stromal Cell Therapeutic Delivery: Translational Challenges to Clinical Application. Front Immunol (2019) 10:1645:1645. 10.3389/fimmu.2019.01645 31417542 PMC6685059

[107] Moll G Drzeniek N Kamhieh-Milz J Geissler S Volk H-D Reinke P MSC Therapies for COVID-19: Importance of Patient Coagulopathy, Thromboprophylaxis, Cell Product Quality and Mode of Delivery for Treatment Safety and Efficacy. Front Immunol (2020) 11:1091:1091. 10.3389/fimmu.2020.01091 32574263 PMC7249852

[108] Uccelli A Moretta L Pistoia V Mesenchymal stem cells in health and disease. Nat Rev Immunol (2008) 8(9):726–36. 10.1038/nri2395 19172693

[109] Le Blanc K Mougiakakos D Multipotent mesenchymal stromal cells and the innate immune system. Nat Rev Immunol (2012) 12(5):383–96. 10.1038/nri3209 22531326

[110] Khosravi M Bidmeshkipour A Moravej A Hojjat-Assari S Naserian S Karimi MH Induction of CD4+CD25+Foxp3+ regulatory T cells by mesenchymal stem cells is associated with RUNX complex factors. Immunol Res (2018) 66(1):207–18. 10.1007/s12026-017-8973-4 29143918

[111] Beldi G Khosravi M Abdelgawad ME Salomon BL Uzan G Haouas H TNFα/TNFR2 signaling pathway: an active immune checkpoint for mesenchymal stem cell immunoregulatory function. Stem Cell Res Ther (2020) 11(1):281. 10.1186/s13287-020-01740-5 32669116 PMC7364521

[112] Khosravi M Karimi MH Hossein Aghdaie M Kalani M Naserian S Bidmeshkipour A Mesenchymal stem cells can induce regulatory T cells via modulating miR-126a but not miR-10a. Gene (2017) 627:327–36. 10.1016/j.gene.2017.06.012 28600182

[113] Khosravi M Bidmeshkipour A Cohen JL Moravej A Hojjat-Assari S Naserian S Induction of CD4+CD25+FOXP3+ regulatory T cells by mesenchymal stem cells is associated with modulation of ubiquitination factors and TSDR demethylation. Stem Cell Res Ther (2018) 9(1):273. 10.1186/s13287-018-0991-1 30359308 PMC6203284

[114] Negi N Griffin MD Effects of mesenchymal stromal cells on regulatory T cells: Current understanding and clinical relevance: MSC effects on T-reg. Stem Cells (2020) 38(5):596-605. 10.1002/stem.3151. Published online February 3, 2020. 31995249 PMC7217190

[115] Vasandan AB Jahnavi S Shashank C Prasad P Kumar A Prasanna SJ Human Mesenchymal stem cells program macrophage plasticity by altering their metabolic status via a PGE 2 -dependent mechanism. Sci Rep (2016) 6(1):38308. 10.1038/srep38308 27910911 PMC5133610

[116] Sudres M Norol F Trenado A Grégoire S Charlotte F Levacher B Bone marrow mesenchymal stem cells suppress lymphocyte proliferation in vitro but fail to prevent graft-versus-host disease in mice. J Immunol (2006) 176(12):7761–7. 10.4049/jimmunol.176.12.7761 16751424

[117] Baron F Lechanteur C Willems E Bruck F Baudoux E Seidel L Cotransplantation of mesenchymal stem cells might prevent death from graft-versus-host disease (GVHD) without abrogating graft-versus-tumor effects after HLA-mismatched allogeneic transplantation following nonmyeloablative conditioning. Biol Blood Marrow Transplant (2010) 16(6):838–47. 10.1016/j.bbmt.2010.01.011 20109568

[118] Ning H Yang F Jiang M Hu L Feng K Zhang J The correlation between cotransplantation of mesenchymal stem cells and higher recurrence rate in hematologic malignancy patients: outcome of a pilot clinical study. Leukemia (2008) 22(3):593–9. 10.1038/sj.leu.2405090 18185520

[119] Moritani K Miyawaki R Tokuda K Ochi F Eguchi-Ishimae M Tauchi H Mesenchymal Stem Cell Therapy Overcomes Steroid Resistance in Severe Gastrointestinal Acute Graft-Versus-Host Disease. Case Rep Transplant (2019) 2019:7890673. 10.1155/2019/7890673 31263624 PMC6556259

[120] Théry C Zitvogel L Amigorena S Exosomes: composition, biogenesis and function. Nat Rev Immunol (2002) 2(8):569–79. 10.1038/nri855 12154376

[121] Balaj L Lessard R Dai L Cho Y-J Pomeroy SL Breakefield XO Tumour microvesicles contain retrotransposon elements and amplified oncogene sequences. Nat Commun (2011) 2:180. 10.1038/ncomms1180 21285958 PMC3040683

[122] Zhang B Yeo RWY Lai RC Sim EWK Chin KC Lim SK Mesenchymal stromal cell exosome-enhanced regulatory T-cell production through an antigen-presenting cell-mediated pathway. Cytotherapy (2018) 20(5):687–96. 10.1016/j.jcyt.2018.02.372 29622483

[123] Balbi C Piccoli M Barile L Papait A Armirotti A Principi E First Characterization of Human Amniotic Fluid Stem Cell Extracellular Vesicles as a Powerful Paracrine Tool Endowed with Regenerative Potential. Stem Cells Transl Med (2017) 6(5):1340–55. 10.1002/sctm.16-0297 PMC5442724 28271621

[124] Cosenza S Ruiz M Toupet K Jorgensen C Noël D Mesenchymal stem cells derived exosomes and microparticles protect cartilage and bone from degradation in osteoarthritis. Sci Rep (2017) 7(1):16214. 10.1038/s41598-017-15376-8 29176667 PMC5701135

[125] Cao L Xu H Wang G Liu M Tian D Yuan Z Extracellular vesicles derived from bone marrow mesenchymal stem cells attenuate dextran sodium sulfate-induced ulcerative colitis by promoting M2 macrophage polarization. Int Immunopharmacol (2019) 72:264–74. 10.1016/j.intimp.2019.04.020 31005036

[126] Lai P Weng J Guo L Chen X Du X Novel insights into MSC-EVs therapy for immune diseases. Biomarker Res (2019) 7(1):6. 10.1186/s40364-019-0156-0 PMC6423844 30923617

[127] Fujii S Miura Y Fujishiro A Shindo T Shimazu Y Hirai H Graft-Versus-Host Disease Amelioration by Human Bone Marrow Mesenchymal Stromal/Stem Cell-Derived Extracellular Vesicles Is Associated with Peripheral Preservation of Naive T Cell Populations. Stem Cells (2018) 36(3):434–45. 10.1002/stem.2759 29239062

[128] Kordelas L Rebmann V Ludwig A-K Radtke S Ruesing J Doeppner TR MSC-derived exosomes: a novel tool to treat therapy-refractory graft-versus-host disease. Leukemia (2014) 28(4):970–3. 10.1038/leu.2014.41 24445866

[129] Haniffa M Ginhoux F Wang X-N Bigley V Abel M Dimmick I Differential rates of replacement of human dermal dendritic cells and macrophages during hematopoietic stem cell transplantation. J Exp Med (2009) 206(2):371–85. 10.1084/jem.20081633 PMC2646566 19171766

[130] Chitu V Stanley ER Colony-stimulating factor-1 in immunity and inflammation. Curr Opin Immunol (2006) 18(1):39–48. 10.1016/j.coi.2005.11.006 16337366

[131] Hashimoto D Chow A Greter M Saenger Y Kwan W-H Leboeuf M Pretransplant CSF-1 therapy expands recipient macrophages and ameliorates GVHD after allogeneic hematopoietic cell transplantation. J Exp Med (2011) 208(5):1069–82. 10.1084/jem.20101709 PMC3092347 21536742

[132] Hong Y-Q Wan B Li X-F Macrophage regulation of graft-vs-host disease. World J Clin Cases (2020) 8(10):1793–805. 10.12998/wjcc.v8.i10.1793 PMC7262718 32518770

[133] Wen Q Kong Y Zhao H-Y Zhang Y-Y Han T-T Wang Y G-CSF-induced macrophage polarization and mobilization may prevent acute graft-versus-host disease after allogeneic hematopoietic stem cell transplantation. Bone Marrow Transplant (2019) 54(9):1419–33. 10.1038/s41409-019-0449-9 30683906

[134] Cheng Q Ma S Lin D Mei Y Gong H Lei L The S1P1 receptor-selective agonist CYM-5442 reduces the severity of acute GVHD by inhibiting macrophage recruitment. Cell Mol Immunol (2015) 12(6):681–91. 10.1038/cmi.2014.59 PMC4716627 25088224

[135] Bouchlaka MN Moffitt AB Kim J Kink JA Bloom DD Love C Human Mesenchymal Stem Cell-Educated Macrophages Are a Distinct High IL-6-Producing Subset that Confer Protection in Graft-versus-Host-Disease and Radiation Injury Models. Biol Blood Marrow Transplant (2017) 23(6):897–905. 10.1016/j.bbmt.2017.02.018 28257800 PMC5499382

[136] Song W-J Li Q Ryu M-O Nam A An J-H Jung YC Canine adipose tissue-derived mesenchymal stem cells pre-treated with TNF-alpha enhance immunomodulatory effects in inflammatory bowel disease in mice. Res Vet Sci (2019) 125:176–84. 10.1016/j.rvsc.2019.06.012 PMC7111869 31247473

[137] Grygorowicz MA Biernacka M Bujko M Nowak E Rymkiewicz G Paszkiewicz-Kozik E Human regulatory T cells suppress proliferation of B lymphoma cells. Leuk Lymphoma (2016) 57(8):1903–20. 10.3109/10428194.2015.1121260 26758248

[138] Janssens W Carlier V Wu B VanderElst L Jacquemin MG Saint-Remy J-MR CD4+CD25+ T cells lyse antigen-presenting B cells by Fas-Fas ligand interaction in an epitope-specific manner. J Immunol (2003) 171(9):4604–12. 10.4049/jimmunol.171.9.4604 14568934

[139] Piccirillo CA Shevach EM Naturally-occurring CD4+CD25+ immunoregulatory T cells: central players in the arena of peripheral tolerance. Semin Immunol (2004) 16(2):81–8. 10.1016/j.smim.2003.12.003 15036231

[140] Serra P Amrani A Yamanouchi J Han B Thiessen S Utsugi T CD40 ligation releases immature dendritic cells from the control of regulatory CD4+CD25+ T cells. Immunity (2003) 19(6):877–89. 10.1016/S1074-7613(03)00327-3 14670304

[141] Alho AC Kim HT Chammas MJ Reynolds CG Matos TR Forcade E Unbalanced recovery of regulatory and effector T cells after allogeneic stem cell transplantation contributes to chronic GVHD. Blood (2016) 127(5):646–57. 10.1182/blood-2015-10-672345 PMC4742552 26670634

[142] Buckner JH Mechanisms of impaired regulation by CD4(+)CD25(+)FOXP3(+) regulatory T cells in human autoimmune diseases. Nat Rev Immunol (2010) 10(12):849–59. 10.1038/nri2889 PMC3046807 21107346

[143] Cohen JL Trenado A Vasey D Klatzmann D Salomon BL CD4(+)CD25(+) immunoregulatory T Cells: new therapeutics for graft-versus-host disease. J Exp Med (2002) 196(3):401–6. 10.1084/jem.20020090 PMC2193933 12163568

[144] Hoffmann P Ermann J Edinger M Fathman CG Strober S Donor-type CD4(+)CD25(+) regulatory T cells suppress lethal acute graft-versus-host disease after allogeneic bone marrow transplantation. J Exp Med (2002) 196(3):389–99. 10.1084/jem.20020399 PMC2193938 12163567

[145] Taylor PA Lees CJ Blazar BR The infusion of ex vivo activated and expanded CD4(+)CD25(+) immune regulatory cells inhibits graft-versus-host disease lethality. Blood (2002) 99(10):3493–9. 10.1182/blood.V99.10.3493 11986199

[146] Edinger M Hoffmann P Ermann J Drago K Fathman CG Strober S CD4+CD25+ regulatory T cells preserve graft-versus-tumor activity while inhibiting graft-versus-host disease after bone marrow transplantation. Nat Med (2003) 9(9):1144–50. 10.1038/nm915 12925844

[147] Gaidot A Landau DA Martin GH Bonduelle O Grinberg-Bleyer Y Matheoud D Immune reconstitution is preserved in hematopoietic stem cell transplantation coadministered with regulatory T cells for GVHD prevention. Blood (2011) 117(10):2975–83. 10.1182/blood-2010-08-299974 21193693

[148] Brunstein CG Miller JS McKenna DH Hippen KL DeFor TE Sumstad D Umbilical cord blood-derived T regulatory cells to prevent GVHD: kinetics, toxicity profile, and clinical effect. Blood (2016) 127(8):1044–51. 10.1182/blood-2015-06-653667 PMC4768428 26563133

[149] Sawitzki B Harden PN Reinke P Moreau A Hutchinson JA Game DS Regulatory cell therapy in kidney transplantation (The ONE Study): a harmonised design and analysis of seven non-randomised, single-arm, phase 1/2A trials. Lancet (2020) 395(10237):1627–39. 10.1016/S0140-6736(20)30167-7 PMC7613154 32446407

[150] Bluestone JA Tang Q Treg cells-the next frontier of cell therapy. Science (2018) 362(6411):154–5. 10.1126/science.aau2688 30309932

[151] Singer BD King LS D’Alessio FR Regulatory T cells as immunotherapy. Front Immunol (2014) 5:46:46. 10.3389/fimmu.2014.00046 24575095 PMC3920065

[152] Trenado A Charlotte F Fisson S Yagello M Klatzmann D Salomon BL Recipient-type specific CD4+CD25+ regulatory T cells favor immune reconstitution and control graft-versus-host disease while maintaining graft-versus-leukemia. J Clin Invest (2003) 112(11):1688–96. 10.1172/JCI17702 PMC281639 14660744

[153] Trenado A Fisson S Braunberger E Klatzmann D Salomon BL Cohen JL Ex vivo selection of recipient-type alloantigen-specific CD4(+)CD25(+) immunoregulatory T cells for the control of graft-versus-host disease after allogeneic hematopoietic stem-cell transplantation. Transplantation (2004) 77(1 Suppl):S32–34. 10.1097/01.TP.0000106470.07410.CA 14726768

[154] Trenado A Sudres M Tang Q Maury S Charlotte F Grégoire S Ex vivo-expanded CD4+CD25+ immunoregulatory T cells prevent graft-versus-host-disease by inhibiting activation/differentiation of pathogenic T cells. J Immunol (2006) 176(2):1266–73. 10.4049/jimmunol.176.2.1266 16394018

[155] Martin GH Grégoire S Landau DA Pilon C Grinberg-Bleyer Y Charlotte F In vivo activation of transferred regulatory T cells specific for third-party exogenous antigen controls GVH disease in mice. Eur J Immunol (2013) 43(9):2263–72. 10.1002/eji.201343449 PMC4738555 23765389

[156] Naserian S Leclerc M Thiolat A Pilon C Le Bret C Belkacemi Y Simple, Reproducible, and Efficient Clinical Grading System for Murine Models of Acute Graft-versus-Host Disease. Front Immunol (2018) 9:10:10. 10.3389/fimmu.2018.00010 29403494 PMC5786520

[157] Leclerc M Naserian S Pilon C Thiolat A Martin GH Pouchy C Control of GVHD by regulatory T cells depends on TNF produced by T cells and TNFR2 expressed by regulatory T cells. Blood (2016) 128(12):1651–9. 10.1182/blood-2016-02-700849 27506541

[158] Brunstein CG Miller JS Cao Q McKenna DH Hippen KL Curtsinger J Infusion of ex vivo expanded T regulatory cells in adults transplanted with umbilical cord blood: safety profile and detection kinetics. Blood (2011) 117(3):1061–70. 10.1182/blood-2010-07-293795 PMC3035067 20952687

[159] Di Ianni M Falzetti F Carotti A Terenzi A Castellino F Bonifacio E Tregs prevent GVHD and promote immune reconstitution in HLA-haploidentical transplantation. Blood (2011) 117(14):3921–8. 10.1182/blood-2010-10-311894 21292771

[160] Chang C-C Satwani P Oberfield N Vlad G Simpson LL Cairo MS Increased induction of allogeneic-specific cord blood CD4+CD25+ regulatory T (Treg) cells: a comparative study of naïve and antigenic-specific cord blood Treg cells. Exp Hematol (2005) 33(12):1508–20. 10.1016/j.exphem.2005.09.002 16338494

[161] Porter SB Liu B Rogosheske J Levine BL June CH Kohl VK Suppressor function of umbilical cord blood-derived CD4+CD25+ T-regulatory cells exposed to graft-versus-host disease drugs. Transplantation (2006) 82(1):23–9. 10.1097/01.tp.0000225824.48931.af 16861937

[162] Ahmadzadeh M Rosenberg SA IL-2 administration increases CD4+ CD25(hi) Foxp3+ regulatory T cells in cancer patients. Blood (2006) 107(6):2409–14. 10.1182/blood-2005-06-2399 PMC1473973 16304057

[163] Zhang H Chua KS Guimond M Kapoor V Brown MV Fleisher TA Lymphopenia and interleukin-2 therapy alter homeostasis of CD4+CD25+ regulatory T cells. Nat Med (2005) 11(11):1238–43. 10.1038/nm1312 16227988

[164] Zorn E Nelson EA Mohseni M Porcheray F Kim H Litsa D IL-2 regulates FOXP3 expression in human CD4+CD25+ regulatory T cells through a STAT-dependent mechanism and induces the expansion of these cells in vivo. Blood (2006) 108(5):1571–9. 10.1182/blood-2006-02-004747 PMC1895505 16645171

[165] Kennedy-Nasser AA Ku S Castillo-Caro P Hazrat Y Wu M-F Liu H Ultra low-dose IL-2 for GVHD prophylaxis after allogeneic hematopoietic stem cell transplantation mediates expansion of regulatory T cells without diminishing antiviral and antileukemic activity. Clin Cancer Res (2014) 20(8):2215–25. 10.1158/1078-0432.CCR-13-3205 PMC3989436 24573552

[166] Koreth J Matsuoka K Kim HT McDonough SM Bindra B Alyea EP Interleukin-2 and regulatory T cells in graft-versus-host disease. N Engl J Med (2011) 365(22):2055–66. 10.1056/NEJMoa1108188 PMC3727432 22129252

[167] Koreth J Kim HT Jones KT Lange PB Reynolds CG Chammas MJ Efficacy, durability, and response predictors of low-dose interleukin-2 therapy for chronic graft-versus-host disease. Blood (2016) 128(1):130–7. 10.1182/blood-2016-02-702852 PMC4937358 27073224

[168] Whangbo JS Kim HT Mirkovic N Leonard L Poryanda S Silverstein S Dose-escalated interleukin-2 therapy for refractory chronic graft-versus-host disease in adults and children. Blood Adv (2019) 3(17):2550–61. 10.1182/bloodadvances.2019000631 PMC6737411 31471324

[169] Diefenbach A Colonna M Koyasu S Development, differentiation, and diversity of innate lymphoid cells. Immunity (2014) 41(3):354–65. 10.1016/j.immuni.2014.09.005 PMC4171710 25238093

[170] Seillet C Belz GT Huntington ND Development, Homeostasis, and Heterogeneity of NK Cells and ILC1. Curr Top Microbiol Immunol (2016) 395:37–61. 10.1007/82_2015_474 26305047

[171] Halim TYF Group 2 innate lymphoid cells in disease. Int Immunol (2016) 28(1):13–22. 10.1093/intimm/dxv050 26306498 PMC5891987

[172] Montaldo E Juelke K Romagnani C Group 3 innate lymphoid cells (ILC3s): Origin, differentiation, and plasticity in humans and mice. Eur J Immunol (2015) 45(8):2171–82. 10.1002/eji.201545598 26031799

[173] McKenzie ANJ Spits H Eberl G Innate lymphoid cells in inflammation and immunity. Immunity (2014) 41(3):366–74. 10.1016/j.immuni.2014.09.006 25238094

[174] Sonnenberg GF Artis D Innate lymphoid cells in the initiation, regulation and resolution of inflammation. Nat Med (2015) 21(7):698–708. 10.1038/nm.3892 26121198 PMC4869856

[175] Hanash AM Dudakov JA Hua G O'Connor MH Young LF Singer NV Interleukin-22 protects intestinal stem cells from immune-mediated tissue damage and regulates sensitivity to graft versus host disease. Immunity (2012) 37(2):339–50. 10.1016/j.immuni.2012.05.028 PMC3477611 22921121

[176] Lindemans CA Calafiore M Mertelsmann AM O'Connor MH Dudakov JA Jenq RR Interleukin-22 promotes intestinal-stem-cell-mediated epithelial regeneration. Nature (2015) 528(7583):560–4. 10.1038/nature16460 PMC4720437 26649819

[177] Dudakov JA Hanash AM Jenq RR Young LF Ghosh A Singer NV Interleukin-22 drives endogenous thymic regeneration in mice. Science (2012) 336(6077):91–5. 10.1126/science.1218004 PMC3616391 22383805

[178] Konya V Mjösberg J Innate lymphoid cells in graft-versus-host disease. Am J Transplant (2015) 15(11):2795–801. 10.1111/ajt.13394 PMC4973689 26228632

[179] Monticelli LA Sonnenberg GF Abt MC Alenghat T Ziegler CGK Doering TA Innate lymphoid cells promote lung-tissue homeostasis after infection with influenza virus. Nat Immunol (2011) 12(11):1045–54. 10.1031/ni.2131 PMC3320042 21946417

[180] Munneke JM Björklund AT Mjösberg JM Garming-Legert K Bernink JH Blom B Activated innate lymphoid cells are associated with a reduced susceptibility to graft-versus-host disease. Blood (2014) 124(5):812–21. 10.1182/blood-2013-11-536888 24855210

[181] Couturier M Lamarthée B Arbez J Renauld J-C Bossard C Malard F IL-22 deficiency in donor T cells attenuates murine acute graft-versus-host disease mortality while sparing the graft-versus-leukemia effect. Leukemia (2013) 27(7):1527–37. 10.1038/leu.2013.39 23399894

[182] Shao L Pan S Zhang QP Jamal M Chen LH Yin Q An Essential Role of Innate Lymphoid Cells in the Pathophysiology of Graft-vs.-Host Disease. Front Immunol (2019) 10:1233. 10.3389/fimmu.2019.01233 31244831 PMC6563595

[183] Kronenberg M Toward an understanding of NKT cell biology: progress and paradoxes. Annu Rev Immunol (2005) 23:877–900. 10.1146/annurev.immunol.23.021704.115742 15771592

[184] Lisbonne M Leite-de-Moraes MC Invariant Valpha14 NKT lymphocytes: a double-edged immuno-regulatory T cell population. Eur Cytokine Netw (2003) 14(1):4–14. 10.1146/annurev.immunol.23.021704.115742 12799208

[185] Leite-De-Moraes MC Moreau G Arnould A Machavoine F Garcia C Papiernik M IL-4-producing NK T cells are biased towards IFN-gamma production by IL-12. Influence of the microenvironment on the functional capacities of NK T cells. Eur J Immunol (1998) 28(5):1507–15. 10.1002/(SICI)1521-4141(199805)28:05<1507::AID-IMMU1507>3.0.CO;2-F 9603455

[186] Leite-de-Moraes MC Lisbonne M Arnould A Machavoine F Herbelin A Dy M Ligand-activated natural killer T lymphocytes promptly produce IL-3 and GM-CSF in vivo: relevance to peripheral myeloid recruitment. Eur J Immunol (2002) 32(7):1897–904. 10.1002/1521-4141(200207)32:7<1897::AID-IMMU1897>3.0.CO;2-Y 12115609

[187] Lisbonne M Hachem P Tonanny M-B Fourneau J-M Sidobre S Kronenberg M In vivo activation of invariant V alpha 14 natural killer T cells by alpha-galactosylceramide sequentially induces Fas-dependent and -independent cytotoxicity. Eur J Immunol (2004) 34(5):1381–8. 10.1002/eji.200324828 15114671

[188] Morris ES MacDonald KPA Rowe V Banovic T Kuns RD Don ALJ NKT cell-dependent leukemia eradication following stem cell mobilization with potent G-CSF analogs. J Clin Invest (2005) 115(11):3093–103. 10.1172/JCI25249 PMC1253626 16224535

[189] Leveson-Gower DB Olson JA Sega EI Luong RH Baker J Zeiser R Low doses of natural killer T cells provide protection from acute graft-versus-host disease via an IL-4-dependent mechanism. Blood (2011) 117(11):3220–9. 10.1182/blood-2010-08-303008 PMC3317770 21258007

[190] Lowsky R Takahashi T Liu YP Dejbakhsh-Jones S Grumet FC Shizuru JA Protective conditioning for acute graft-versus-host disease. N Engl J Med (2005) 353(13):1321–31. 10.1056/NEJMoa050642 16192477

[191] Rubio M-T Moreira-Teixeira L Bachy E Bouillié M Milpied P Coman T Early posttransplantation donor-derived invariant natural killer T-cell recovery predicts the occurrence of acute graft-versus-host disease and overall survival. Blood (2012) 120(10):2144–54. 10.1182/blood-2012-01-404673 22730537

[192] Chaidos A Patterson S Szydlo R Chaudhry MS Dazzi F Kanfer E Graft invariant natural killer T-cell dose predicts risk of acute graft-versus-host disease in allogeneic hematopoietic stem cell transplantation. Blood (2012) 119(21):5030–6. 10.1182/blood-2011-11-389304 PMC6143158 22371885

[193] Ha G Ferratge S Naserian S Proust R Ponsen A-C Arouche N Circulating endothelial progenitors in vascular repair. J Cell Immunother (2018) 4(1):13–7. 10.1016/j.jocit.2018.09.004

[194] Loisel F Provost B Guihaire J Boulate D Arouche N Amsallem M Autologous endothelial progenitor cell therapy improves right ventricular function in a model of chronic thromboembolic pulmonary hypertension. J Thorac Cardiovasc Surg (2019) 157(2):655–66. 10.1016/j.jtcvs.2018.08.083 30669226

[195] Naserian S Abdelgawad ME Lachaux J Arouche N Loisel F Afshar M Development of Bio-Artificial Micro-Vessels with Immunosuppressive Capacities: A Hope for Future Transplantations and Organoids. Blood (2019) 134(Supplement_1):3610–0. 10.1182/blood-2019-121395

[196] Proust R Ponsen A-C Rouffiac V Schenowitz C Montespan F Ser-Le Roux K Cord blood-endothelial colony forming cells are immunotolerated and participate at post-ischemic angiogenesis in an original dorsal chamber immunocompetent mouse model. Stem Cell Res Ther (2020) 11(1):172. 10.1186/s13287-020-01687-7 32381102 PMC7206734

[197] Naserian S Abdelgawad ME Afshar Bakshloo M Ha G Arouche N Cohen JL The TNF/TNFR2 signaling pathway is a key regulatory factor in endothelial progenitor cell immunosuppressive effect. Cell Commun Signal (2020) 18(1):94. 10.1186/s12964-020-00564-3 32546175 PMC7298859

[198] Barkestani MN Shamdani S Bakshloo MA TNFα priming through its interaction with TNFR2 enhances Endothelial Progenitor Cell immunosuppressive effect: new hope for their widespread clinical application. Research Square [PREPRINT] (Version 2). 10.21203/rs.3.rs-71393/v2+ PMC7784277 33397378

[199] Zeng L Chen C Song G Yan Z Xu S Jia L Infusion of endothelial progenitor cells accelerates hematopoietic and immune reconstitution, and ameliorates the graft-versus-host disease after hematopoietic stem cell transplantation. Cell Biochem Biophys (2012) 64(3):213–22. 10.1007/s12013-012-9387-5 22806343

[200] Yan Z Zeng L Li Z Zhang H Chen W Jia L Bone marrow-derived endothelial progenitor cells promote hematopoietic reconstitution after hematopoietic stem cell transplantation. Transplant Proc (2013) 45(1):427–33. 10.1016/j.transproceed.2012.03.064 23375333

[201] Khoo CP Pozzilli P Alison MR Endothelial progenitor cells and their potential therapeutic applications. Regener Med (2008) 3(6):863–76. 10.2217/17460751.3.6.863 18947309

[202] Zhang Y Song G Pan B Hua J Xu K Zeng L [Recovery of vascular niche in bone marrow by donor derived endothelial progenitor cells after allogeneic bone marrow transplantation in mice]. Zhonghua Xue Ye Xue Za Zhi (2012) 33(8):623–7. 10.2217/17460751.3.6.863 23134854

